# Comprehensive analysis of syndromic hearing loss patients in Japan

**DOI:** 10.1038/s41598-019-47141-4

**Published:** 2019-08-19

**Authors:** Michie Ideura, Shin-ya Nishio, Hideaki Moteki, Yutaka Takumi, Maiko Miyagawa, Teruyuki Sato, Yumiko Kobayashi, Kenji Ohyama, Kiyoshi Oda, Takamichi Matsui, Tsukasa Ito, Hiroshi Suzumura, Kyoko Nagai, Shuji Izumi, Nobuhiro Nishiyama, Manabu Komori, Kozo Kumakawa, Hidehiko Takeda, Yoko Kishimoto, Satoshi Iwasaki, Sakiko Furutate, Kotaro Ishikawa, Masato Fujioka, Hiroshi Nakanishi, Jun Nakayama, Rie Horie, Yumi Ohta, Yasushi Naito, Mariko Kakudo, Hirofumi Sakaguchi, Yuko Kataoka, Kazuma Sugahara, Naohito Hato, Takashi Nakagawa, Nana Tsuchihashi, Yukihiko Kanda, Chiharu Kihara, Tetsuya Tono, Ikuyo Miyanohara, Akira Ganaha, Shin-ichi Usami

**Affiliations:** 1 Department of Otorhinolaryngology, Shinshu University School of Medicine, Matsumoto, Japan; 2 Department of Hearing Implant Sciences, Shinshu University School of Medicine, Matsumoto, Japan; 3 Department of Otorhinolaryngology, Head & Neck Surgery, Akita University Graduate School of Medicine, Akita, Japan; 4 Department of Otolaryngology - Head and Neck Surgery, Iwate Medical University, Morioka, Japan; 5 Department of Otorhinolaryngology, Tohoku Rosai Hospital, Sendai, Japan; 6 Department of Otolaryngology, Fukushima Medical University, Fukushima, Japan; 7 Department of Otolaryngology, Head and Neck Surgery, Yamagata University Faculty of Medicine, Yamagata, Japan; 8 Department of Pediatrics, Dokkyo Medical University School of Medicine, Mibu, Japan; 9 TAKASAKI Ear Nose & Throat Clinic, Takasaki, Japan; 10 Department of Otolaryngology Head and Neck Surgery, Niigata University Graduate School of Medical and Dental Sciences, Niigata, Japan; 11 Department of Otorhinolaryngology - Head and Neck Surgery, Tokyo Medical University, Tokyo, Japan; 12 Department of Otorhinolaryngology, The Jikei University School of Medicine, Tokyo, Japan; 13 Department of Otorhinolaryngology, Toranomon Hospital, Tokyo, Japan; 14 Department of Medical Genetics and Genomics, Kitasato University Graduate School of Medical Sciences, Sagamihara, Japan; 15 Department of Otorhinolaryngology, International University of Health and Welfare Mita Hospital, Tokyo, Japan; 16 Department of Otolaryngology, National Rehabilitation Center for Persons with Disabilities, Tokorozawa, Saitama Japan; 17 Department of Otorhinolaryngology - Head and Neck Surgery, Keio University School of Medicine, Tokyo, Japan; 18 Department of Otorhinolaryngology/Head and Neck Surgery, Hamamatsu University School of Medicine, Hamamatsu, Japan; 19 Department of Otorhinolaryngology, Shiga University School of Medical Science, Otsu, Japan; 20 Department of Otolaryngology, Shiga Medical Center for Children, Moriyama, Shiga, Japan; 21 Department of Otorhinolaryngology - Head and Neck Surgery, Osaka University Graduate School of Medicine, Suita, Japan; 22 Departments of Otolaryngology - Head and Neck Surgery, Kobe City Medical Center General Hospital, Kobe, Japan; 23 Department of clinical genetics, Hyogo College of Medicine, Nishinomiya, Japan; 24 Department of Otorhinolaryngology - Head and Neck Surgery, Kyoto Prefectural University of Medicine, Kyoto, Japan; 25 Department of Otolaryngology - Head and Neck Surgery, Okayama University Graduate School of Medicine, Dentistry and Pharmaceutical Sciences, Okayama, Japan; 26 Department of Otolaryngology, Yamaguchi University Graduate School of Medicine, Ube, Japan; 27 Department of Otorhinolaryngology, Head and Neck surgery Ehime University, School of Medicine, Toon, Japan; 28 Department of Otorhinolaryngology, Graduate School of Medical Sciences, Kyushu University, Fukuoka, Japan; 29 Kanda ENT Clinic, Nagasaki Bell Hearing center, Nagasaki, Japan; 30 Department of Otolaryngology-Head and Neck Surgery, Nagasaki University Graduate School of Biomedical Sciences, Nagasaki, Japan; 31 Department of Otolaryngology Head and Neck Surgery, Faculty of Medicine, University of Miyazaki, Miyazaki, Japan; 32 Department of Otolaryngology - Head and Neck Surgery, Kagoshima University Graduate School of Medical and Dental Sciences, Kagoshima, Japan; 33 Department of Otorhinolaryngology, Head and Neck Surgery, Graduate School of Medicine, University of the Ryukyus, Nishihara-cho, Japan

**Keywords:** Genetics research, Genetic testing

## Abstract

More than 400 syndromes associated with hearing loss and other symptoms have been described, corresponding to 30% of cases of hereditary hearing loss. In this study we aimed to clarify the mutation spectrum of syndromic hearing loss patients in Japan by using next-generation sequencing analysis with a multiple syndromic targeted resequencing panel (36 target genes). We analyzed single nucleotide variants, small insertions, deletions and copy number variations in the target genes. We enrolled 140 patients with any of 14 syndromes (BOR syndrome, Waardenburg syndrome, osteogenesis imperfecta, spondyloepiphyseal dysplasia congenita, Stickler syndrome, CHARGE syndrome, Jervell and Lange-Nielsen syndrome, Pendred syndrome, Klippel-Feil syndrome, Alport syndrome, Norrie disease, Treacher-Collins syndrome, Perrault syndrome and auditory neuropathy with optic atrophy) and identified the causative variants in 56% of the patients. This analysis could identify the causative variants in syndromic hearing loss patients in a short time with a high diagnostic rate. In addition, it was useful for the analysis of the cases who only partially fulfilled the diagnostic criteria.

## Introduction

Congenital hearing loss is one of the most common sensory disorders, affecting one out of 500–1000 newborns. Over half of the cases of congenital or early onset sensorineural hearing loss are estimated to be caused by genetic factors^1^, with 30% of these hereditary hearing loss patients affected by various syndromes. More than 400 syndromes associated with hearing loss and other symptoms have been described^2^.

The most commonly observed syndromes in clinical settings include Pendred syndrome, BOR syndrome, Waardenburg syndrome, osteogenesis imperfecta, Stickler syndrome, spondyloepiphyseal dysplasia congenita, CHARGE syndrome, Klippel-Feil syndrome, Alport syndrome, Treacher-Collins syndrome, Jervell Lange-Nielsen syndrome, Perrault syndrome, Norrie disease, and auditory neuropathy with optic atrophy. The clinical characteristics and responsible genes for these 14 syndromes are summarized in Table 1.

**Table 1:** The clinical characteristics and responsible genes for 14 types of syndromic hearing loss.

Syndrome	OMIM#	Prevalence	Gene and inheritance	Clinical features	Reference
Branchio-oto-renal (BOR) syndrome	113650, 602588, 608389, 610896	1:40,000	*EYA1* (AD), *SIX1* (AD), *SIX5* (AD)	hearing loss, branchial anomalies, preauricular pits, renal anomalies, anomalies of the external, middle, inner ear, and others	^3, 4^
Waardenburg syndrome (WS) type 1	193500	1:20,000–40,000 for all types of WS	*PAX3* (AD)	hearing loss, pigmentation disturbances of the hair, skin and eyes, dystopia canthorum	^5– 9^
Waardenburg syndrome (WS) type 2	193510, 608890, 611584		*MITF* (AD), *SNAI2* (AR), *SOX10* (AD), *EDNRB* (AD),	hearing loss, pigmentation disturbances of the hair, skin and eyes	^5– 9^
Waardenburg syndrome (WS) type 3	148820		*PAX3* (AD)	hearing loss, pigmentation disturbances of the hair, skin and eyes, dystopia canthorum, upper limb abnormalities	^5– 9^
Waardenburg syndrome (WS) type 4	277580, 613265, 613266		*EDNRB* (AD/AR), *EDN3* (AD/AR), *SOX10* (AD),	hearing loss, pigmentation disturbances of the hair, skin and eyes, Hirschsprung disease	^5– 9^
Osteogenesis imperfecta	166200, 166210, 259420, 166220	1:15,000–20,000	*COL1A1* (AD), *COL1A2* (AD)	hearing loss, multiple bone fractures, blue sclera, otosclerosis	^10^
Spondyloepiphyseal displasia congenita	183900	unknown	*COL2A1* (AD)	hearing loss, short stature, abnormal epiphyses, flattened body	^11^
Stickler syndrome	108300, 604841, 614134, 614284	1:7,500–9,000	*COL2A1* (*AD*), *COL11A1* (AD), *COL9A1* (AR), *COL9A2* (AR), *COL9A3* (AR)	hearing loss, cleft palate, midfacial hypoplasia, arthritis, eye sympton (myopia, retinal retachment)	^12– 16^
Stickler syndrome (non-ocular type)		unknown	*COL11A2* (AD/AR)	hearing loss, cleft palate, midfacial hypoplasia, arthritis	^12, 17^
Alport syndrome	301050, 203780, 104200	1:50,000	*COL4A5* (XLD), *COL4A3* (AD/AR), *COL4A4* (AR)	hearing loss, eye sympton, renal dysfunction	^18– 20^
CHARGE syndrome	214800	1:8,500–10,000	*CHD7* (AD), *SEMA3E* (AD)	hearing loss/ear anomalies, coloboma, heart defect, choanal atresia, retarded growth and development, genital hypoplasia	^21– 24^
Jervell and Lange-Nielsen syndrome	220400, 612347	1:200,000	*KCNQ1* (AR), *KCNE1* (AR)	hearing loss, a long QT interval with torsade de pointes on an electrocardiogram	^25, 26^
Pendred syndrome	274600	1:10,000–13,000	*SLC26A4* (AR)	hearing loss, goiter, enlarged vestibular aqueduct	^27^
Klippel-Feil syndrome	118100, 214300	1:40,000–42,000	*GDF6* (AD), *MEOX1* (AR), *GDF3* (AD), *MYO18B* (AR)	hearing loss, short neck (fusion of cevicalvertebrae), low posterior hairline	^59^
Auditory neuropathy with optic atrophy		unknown	*OPA1* (AD)	hearing loss, visual impairment (optic atrophy)	^60^
Treacher-Collins syndrome	154500, 248390	1:50,000	*TCOF1* (AD), *POLR1D* (AD/AR), *POLR1C* (AR)	hearing loss malformations of ear, eye, and mandibula	
Norrie disease	310600	unknown	*NDP* (AR)	hearing loss, eye symptoms (pseudoglioma, blindness), mental retardation	
Perrault syndrome	233400, 614926, 614129, 615300	unknown	*HSD17B4* (AR), *HARS2* (AR), *CLPP* (AR), *LARS2* (AR), *TWNK* (AR), *ERAL1* (AR)	hearing loss, ovarian dysgenesis (in females)	

AD: Autosomal dominant, AR: Autosomal recessive, XLD: X-linked dominant. Responsible genes, prevalence, inheritance and clinical feature informations were obtained from OMIM database (https://www.omim.org), GeneReviews^®^, StatPearls and each reference.

Branchio-Oto-Renal (BOR) syndrome (OMIM#113650 and #610896) or Branchio-Oto (BO) syndrome (OMIM#602588 and 608389) is characterized by the association of the branchial arch, external ear anomalies, hearing impairment and renal anomalies. BO/BOR syndrome is observed in one out of 40,000 children, and in 2% of profoundly deaf children^3,4^.

Waardenburg syndrome (WS1 OMIM#193500, WS2 OMIM#193510, #608890 and #611584, WS3 OMIM#148820, WS4 OMIM#277580, #613265 and #613266) is characterized by varying degrees of hearing impairment and pigmentation disturbances in the hair, skin and eyes^5,6^. WS is classified into four types based on clinical findings. The frequency of WS is 1/20,000–40,000 newborns^5–9^. Osteogenesis imperfecta type 1 (OMIM#166200) is an autosomal dominant inheritance disorder characterized by fractures with minimal or no trauma, blue sclera, hearing loss and otosclerosis^10^.

Spondyloepiphyseal dysplasia congenita (OMIM#183900) is an autosomal dominantly inherited chondrodysplasia characterized by a disproportionately short stature (short trunk), abnormal epiphyses and flattened vertebral bodies^11^.

Stickler syndrome (OMIM#108300, #604841, #614134 and #614284) is an inherited connective tissue disorder associated with myopia, retinal detachment, cleft palate, midfacial hypoplasia, arthritis and hearing impairment^12–17^. Alport syndrome (OMIM#301050, #203780 and #104200) is a progressive disease associated with glomerulonephritis, sensorineural hearing loss, and ocular complications caused by abnormalities in type IV collagen^18–20^.

CHARGE syndrome (OMIM#214800) is an autosomal dominant disorder characterized by congenital multiple anomalies (coloboma, heart defect, choanal atresia, retarded growth and development, genital hypoplasia and ear anomalies/deafness)^21–24^.

Jervell and Lange-Nielsen syndrome (OMIM#220400 and #612347) is a rare autosomal recessive cardio-auditory disorder characterized by congenital profound bilateral sensorineural hearing loss and a long QT interval with arrhythmia (torsade de pointes)^25,26^

Pendred syndrome (OMIM#274600) is an autosomal recessive disorder characterized by congenital hearing loss, goiter, and enlarged vestibular aqueduct^27^.

In this study, we conducted a comprehensive analysis of 140 Japanese syndromic hearing loss patients to obtain the mutation spectrums and clinical features by using next-generation sequencing (NGS) analysis with a multiple syndromic targeted resequencing panel.

## Results

As shown in Table 2, we performed NGS analysis of 36 previously reported genes associated with syndromic hearing loss for 140 probands and identified the causative gene variants in 79 probands (56%). The diagnostic rate by syndrome was 32% (19/59) for BOR syndrome, 78% (18/23) for Waardenburg syndrome, 60% (3/5) for osteogenesis imperfecta, 100% (3/3) for Stickler syndrome, and 89% (32/36) for Pendred syndrome. On the other hand, we could not detect any causative gene variants for Klippel-Feil syndrome, Alport syndrome or Norrie disease cases.

**Table 2:** Subjects and diagnostic ratio in this study.

Clinical diagnosis	Probands	Genetic diagnosis	Diagnostic rate
Branchio-oto-renal syndrome	59	*EYA1*: 18 cases, *SIX1*: 1 case	32%
Waardenburg syndrome 1	5	*PAX3*: 2 cases, *MITF*: 1 case, *SOX10*: 1 case	80%
Waardenburg syndrome 2	14	*MITF*: 4 cases, *SOX10*: 5 cases, *EDNRB*: 1 case	71%
Waardenburg syndrome (unclassifiable WS1 or WS2)	2	*PAX3*: 2 cases	100%
Waardenburg syndrome 4	2	*SOX10*: 2 cases	100%
Osteogenesis imperfecta	5	*COL1A1*: 3 cases	60%
Stickler syndrome	3	*COL11A1*: 2 cases, *COL11A2*: 1 case	100%
Spondyloepiphyseal dysplasia congenita	1	*COL2A1*: 1 case	100%
CHARGE syndrome	3	*CHD7*: 1 case	33%
Jervell and Lange-Nielsen syndrome	1	*KCNQ1*(compound heterozygous): 1 case	100%
Pendred syndrome	36	*SLC26A4*(compound heterozygous or homozygous): 32 cases	89%
Klippel-Feil syndrome	3		0%
Alport syndrome	4		0%
Treacher-Collins syndrome	0		NA
Norrie disease	1		0%
Perrault syndrome	0		NA
Auditory neuropathy with optic atrophy	1	*OPA1*: 1 case	100%
Total	140	79	56%

### Mutation spectrum and clinical features of BOR syndrome patients

We conducted genetic analysis of 59 probands with clinical findings of BO/BOR syndrome (16 typical cases, 43 atypical cases, Supplementary Table S1), and identified causative heterozygous variants in 19 probands (diagnostic rate 32%). We identified the causative variants in 12/16 typical cases, but the causative variants were identified in only 7/43 atypical cases. Table 3 summarizes the identified variants and clinical features of the probands and all affected family members for 18 families with *EYA1* mutations, and one family with a *SIX1* mutation. There were no BO/BOR cases caused by the *SIX5* gene variant. Among the 12 *EYA1* variants, 8 were truncating variants (five were nonsense, one was frameshift, two were splice site), three were missense variants and one was a copy number variation (one copy number loss). Four of them were novel variants and 8 of them were previously reported. JHLB4043 had one copy loss detected using NGS read depth data, which seemed to be deleted in all of the *EYA1* gene, confirmed by array Comparative Genomic Hybridization (aCGH). The mutations identified in this study were located in exon 6 to exon 13, and frequently observed in exon 8 and exon 12. Two or more cases carried the same variants (p.R264X, p.R275X, c.867 + 5 G > A, p.R328X and p.R407Q). *EYA1* variants were mainly identified from autosomal dominant families (10/18 cases); however, we also identified variants from 7 sporadic cases. Among them we confirmed *de novo* mutations in four families (Supplementary Fig. S1). The case with a *SIX1* mutation was also caused by *de novo* mutation (Supplementary Fig. S1). In terms of the clinical features of all BO/BOR-affected patients with *EYA1* and *SIX1* gene variants (19 probands and their family members who carried the same variants; 34 patients in total), the most frequent symptom was hearing loss (31/ 32, 97%). Unilateral hearing loss was observed in 2 cases. The most frequent type of hearing loss was moderate mixed hearing loss. Middle and/or inner ear anomalies were observed in 22 of 23 cases who underwent CT imaging (96%). Twenty-seven of 31 cases had preauricular pits (87%), and 14 of 25 cases for whom information was available had branchial anomalies (56%). Renal anomalies, on the other hand, were revealed in only one of 7 cases for whom kidney abnormalities were examined (14%). It is noteworthy that there were only a limited number of cases (7/34) with renal ultrasonographic information available in our cohort, thus the frequencies of renal anomalies may be underestimated. The presence of branchial or renal anomalies was not correlated with the severity of hearing loss. Furthermore, no relationship was found between genotype and clinical findings. As rare symptoms, one patient had hemifacial palsy, and 3 cases had eye symptoms.

**Table 3:** Genetic diagnosis results and clinical features of BO/BOR syndrome patients and family members.

Proband	Family	Type	Nucleotide change	Amino Acid change	Location	Hereditary form	Severity of hearing loss		Preauricular pits	Cervical fistula	Ear marformation			Renal anomaly	Other clinical features	reference
							Rt.ear	Lt.ear			Inner ear	Middle ear	External ear			
JHLB-6679	proband	typical	*EYA1*: c. [489T > G]; [=]	p.[Y163X];[=]	exon 6	AD	moderate	moderate	+	−	+	+	+	−	−	this study
	father	atypical	*EYA1*: c. [489T > G]; [=]	p.[Y163X];[=]	exon 6		moderate	moderate	+	−	NA	NA	NA	−	−	
	brother	NA	*EYA1*: c. [489T > G]; [=]	p.[Y163X];[=]	exon 6		NA	NA	NA	NA	NA	NA	NA	NA	NA	
	mother	unaffected	*EYA1*: c. [=]; [=]				normal	normal	−	−	NA	NA	NA	NA	NA	
JHLB346	proband	typical	*EYA1*: c. [790C > T]; [=]	p.[R264X];[=]	exon 8	AD	mild	normal	+	+	NA	NA	NA	NA	NA	Rickard (2000), Fukuda (2001)
JHLB3868	proband	atypical	*EYA1*: c. [790C > T]; [=]	p.[R264X];[=]	exon 8	AD	moderate	(COR)	+	−	+	NA	NA	NA	−	Rickard (2000), Fukuda (2001)
	grandfather	typical	*EYA1*: c. [790C > T]; [=]	p.[R264X];[=]	exon 8		moderate	profound	+	+	NA	NA	NA	NA	−	
	mother	typical	*EYA1*: c. [790C > T]; [=]	p.[R264X];[=]	exon 8		moderate	moderate	+	+	NA	NA	NA	NA	−	
	father	unaffected	*EYA1*: c. [=]; [=]				NA	NA	−	−	NA	NA	NA	NA	NA	
	grandmother	unaffected	*EYA1*: c. [=]; [=]				NA	NA	−	−	NA	NA	NA	NA	NA	
#4107	proband	typical	*EYA1*: c.[823C > T]; [=]	p.[R275X];[=]	exon 8	AD	moderate	mild	+	NA	+	+	NA	NA	−	Abdelhak (1997), Orten (2008)
	mother	atypical	*EYA1*: c.[823C > T]; [=]	p.[R275X];[=]	exon 8		profound	moderate	NA	+	NA	+	NA	NA	−	
JHLB2279	proband	typical	*EYA1*: c. [823C > T]; [=]	p.[R275X];[=]	exon 8	sporadic (*de novo*)	moderate	moderate	+	+	+	+	NA	−	−	Abdelhak (1997), Orten (2008)
	father	unaffected	*EYA1*: c. [=]; [=]				NA	NA	−	−	NA	NA	NA	NA	NA	
	mother	unaffected	*EYA1*: c. [=]; [=]				NA	NA	−	−	NA	NA	NA	NA	NA	
#371	proband	atypical	*EYA1*: c.[867 + 5G > A];[=]		intron 8	sporadic	mild	profound	+	−	NA	NA	NA	NA	−	Stockley (2008)
JHLB4689	proband	typical	*EYA1*: c.[867+5G > A]; [=]		intron 8	AD	moderate	moderate	+	+	NA	+	NA	NA	−	Stockley (2008)
	brother	atypical	*EYA1*: c.[867+5G > A]; [=]		intron 8		moderate	moderate	+	NA	NA	+	NA	NA	−	
	mother	atypical	*EYA1*: c.[867+5G > A]; [=]		intron 8		mild	mild	+	NA	NA	NA	NA	NA	NA	
	grandmother	atypical	*EYA1*: c.[867+5 G > A]; [=]		intron 8		profound	severe	−	−	NA	NA	NA	NA	NA	
JHLB2062	proband	atypical	*EYA1*: c. [982 C > T]; [=]	p.[R328X];[=]	exon 10	sporadic (*de novo*)	profound	profound	+	NA	+	NA	NA	−	vision Zimpair-ment	Spruijt (2006), Olavarrieta (2008)
	father	unaffected	*EYA1*: c. [=]; [=]				NA	NA	NA	NA	NA	NA	NA	NA	NA	
	mother	unaffected	*EYA1*: c. [=]; [=]				NA	NA	NA	NA	NA	NA	NA	NA	NA	
JHLB2922	proband	typical	*EYA1*: c. [982 C > T]; [=]	p.[R328X];[=]	exon 10	sporadic	moderate	moderate	+	+	+	+	NA	NA	−	Spruijt (2006), Olavarrieta (2008)
JHLB3360	proband	typical	*EYA1*: c. [982C > T]; [=]	p.[R328X];[=]	exon 10	AD	normal	normal	+	+	−	NA	NA	+	−	Spruijt (2006), Olavarrieta (2008)
	mother	atypical	*EYA1*: c. [982C > T]; [=]	p.[R328X];[=]	exon 10		profound	profound	+	NA	NA	NA	NA	−	−	
JHLB975	proband	atypical	*EYA1*: c. [1090C > T]; [=]	p.[Q364X];[=]	exon 11	NA	NA	NA	+	NA	+	NA	NA	NA	NA	this study
JHLB3266	proband	atypical	*EYA1*: c. [1101-1G > A]; [=]		intron 11	AD	severe	severe	−	+	+	NA	NA	NA	amblyopia. hyperopia	Retterer (2016)
	mother	atypical	*EYA1*: c. [1101-1G > A]; [=]		intron 11		severe	profound	−	−	NA	NA	NA	NA	hyperopia	
	uncle	atypical	*EYA1*: c. [1101-1G > A]; [=]		intron 11		severe	profound	−	−	+	NA	NA	NA	NA	
JHLB2645	proband	typical	*EYA1*: c. [1155_1156delAT]; [=]	p. [L385fs];[=]	exon 12	sporadic (*de novo*)	severe	moderate	+	+	+	+	NA	NA	−	this study
	father	unaffected	*EYA1*: c. [=]; [=]				NA	NA	NA	NA	NA	NA	NA	NA	NA	
	mother	unaffected	*EYA1*: c. [=]; [=]				NA	NA	NA	NA	NA	NA	NA	NA	NA	
	sister	unaffected	*EYA1*: c. [=]; [=]				NA	NA	NA	NA	NA	NA	NA	NA	NA	
#4361	proband	typical	*EYA1*: c. [1187A > G]; [=]	p.[D396G];[=]	exon 12	AD	profound	severe	+	+	+	NA	NA	NA	−	Namba (2001)
	daughter	atypical	*EYA1*: c. [1187A > G]; [=]	p.[D396G];[=]	exon 12		normal	profound	+	NA	NA	NA	NA	NA	NA	
#4079	proband	atypical	*EYA1*: c.[1220G > A]; [=]	p.[R407Q];[=]	exon 12	sporadic (*de novo*)	mild	moderate	+	−	+	NA	NA	NA	−	Chang (2004)
	father	unaffected	*EYA1*: c. [=]; [=]				NA	NA	NA	NA	NA	NA	NA	NA	NA	
	mother	unaffected	*EYA1*: c. [=]; [=]				NA	NA	NA	NA	NA	NA	NA	NA	NA	
JHLB2233	proband	typical	*EYA1*: c. [1220G > A]; [=]	p.[R407Q];[=]	exon 12	AD	severe	(COR)	+	−	+	+	−	NA	−	Chang (2004)
	mother	typical	*EYA1*: c. [1220G > A]; [=]	p.[R407Q];[=]	exon 12		profound	moderate	+	+	+	+	+	−	facial palsy	
JHLB2717	proband	atypical	*EYA1*: c. [1376G > C]; [=]	p.[R459P];[=]	exon 13	sporadic	severe	severe	NA	+	+	NA	NA	NA	−	Orten (2008)
JHLB4043	proband	typical	*EYA1*: c.(?_72111486_72268810_?)	CNV		AD	profound	profound	+	−	+	+	+	NA	−	this study
	brother	typical	*EYA1*: c.(?_72111486_72268810_?)	CNV			moderate	moderate	+	+	+	+	−	NA	−	
	father	typical	*EYA1*: c.(?_72111486_72268810_?)	CNV			profound	severe	+	−	+	+	−	NA	−	
JHLB660	proband	typical	*SIX1*: c.[519G > C]; [=]	p. [K173N];[=]	exon 1	sporadic (*de novo*)	profound	(COR)	+	NA	+	+	−	NA	−	Unzaki (2018)
	father	unaffected	*SIX1*: c.[=]; [=]				NA	NA	NA	NA	NA	NA	NA	NA	NA	
	mother	unaffected	*SIX1*: c.[=]; [=]				NA	NA	NA	NA	NA	NA	NA	NA	NA	

AD: Autosomal dominant, COR: Conditioned orientation response audiometry.

The reference cDNA sequences NM_172060 for *EYA1* and NM_005982 for *SIX1*.

### Mutation spectrum and clinical features of Waardenburg syndrome patients

**Figure 1:**
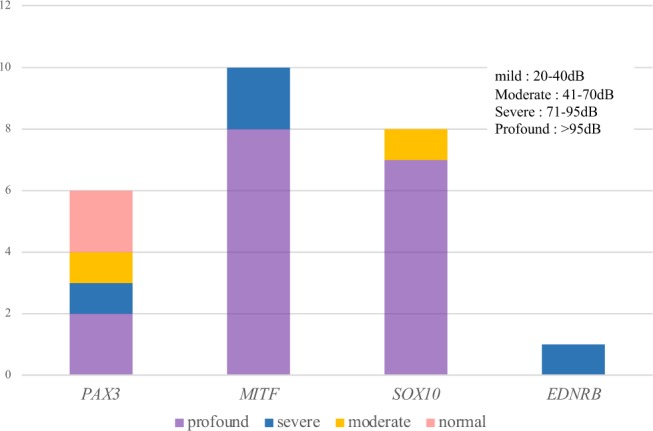
The degree of hearing loss for all family members harboring causative variants. We calculated the hearing threshold in the worse hearing ear. Unilateral hearing loss: *MITF* 3 cases, *EDNRB* 1 case.

We conducted genetic analysis of 23 probands with hearing loss and one or more clinical findings typical of Waardenburg syndrome, and identified the causative heterozygous variants in 18 probands (diagnostic rate 78%). Table 4 and Supplementary Fig. S2 provides a summary of the identified variants and clinical features of probands and all family members (four families with *PAX3* mutations, five families with *MITF* mutations, 8 families with *SOX10* mutations, and one family with a *EDNRB* mutation). No pathogenic variants were found in *SNAI2* or *EDN3*. Most of the identified variants were truncating variants (four were nonsense, 7 were frameshift, one was splice site) and only three cases had missense variants, one each in *PAX3*, *MITF* and *SOX10*. In addition, we also identified three cases with one copy number loss of the *SOX10* gene identified from NGS read depth data and confirmed by aCGH. Thirteen variants were novel and five variants (three *PAX3* mutations, and one each with *MITF* and *SOX10* mutations) were previously reported. Computer prediction scores, allele frequency information and the pathogenicity classification for novel variants are listed in Supplementary Table S2. Autosomal dominant inherited cases were 2/4 in *PAX3* cases, 4/5 in *MITF* cases, 1/8 in *SOX10* cases, and 1/1 in *EDNRB* cases. The other 10 cases were sporadic cases, with confirmed *de novo* mutations in *SOX10* in five cases (Table 4). In terms of the clinical features of the probands and all family members harboring the same causative gene variants (29 patients from 18 families in total), the most frequent symptom was hearing loss (27/29, 93%), followed by heterochromia iridis (23/28, 82%). The severity of hearing loss for each gene is shown in Fig. 1, with the frequency of the profound hearing loss higher in cases with *MITF* and *SOX10* mutations. Two cases with *PAX3* mutations had bilateral normal hearing and three cases with *MITF* mutations had unilateral hearing loss. Only a limited number of patients showed discoloration of the hair and skin: hair discoloration was seen in two cases (with *SOX10* and *MITF* mutations), leukoderma in one case with a *SOX10* mutation, and excessive freckles in three cases with *MITF* mutations. No abnormal musculoskeletal findings were observed in any case. Dystopia canthorum was seen in two cases with *PAX3* mutations, and one each with *MITF* and *SOX10* mutations. The other associated symptoms observed in *SOX10* cases were ptosis (JHLB4270, JHLB4310), developmental delay (JHLB4310) and Asperger syndrome (JHLB3480). In addition, inner ear anomalies, including hypoplasia of the semicircular canal, cochlea, cochlear nerve, and saccular vestibule, were observed. It is suggested that there is no obvious correlation between the type of mutation and its location and the severity of the symptoms. Most of the clinical findings for cases associated with each gene were in agreement with previous reports; however, we identified phenotype-genotype disagreement in two Waardenburg syndrome 1 (WS1) cases (JHLB2469 with a *MITF* mutation and JHLB5132 with a *SOX10* mutation).

**Table 4:** Genetic diagnosis results and clinical features of Waardenburg syndrome patients and family members.

Clinical diagnostic type	Proband	Family	Nucleotidechange	Amino Acid change	Location	Hereditary form	Severity of hearing loss		Dystopia canthorum	Heterochromia	Other clinical features	Reference
							Rt.ear	Lt.ear				
WS1	JHLB1588	proband	*PAX3*: c.[667C > T];[=]	p.[R223X];[=]	exon 5	sporadic	moderate	(COR)	+	+	−	Baldwin (1994)
	JHLB1655	proband	*PAX3*:c. [792+1G > A];[=]		intron 5	AD	profound	(COR)	+	+	−	Wollnik (2003)
		father (U)	*PAX3*:c. [792+1G > A];[=]		intron 5		normal	normal	−	−	−	
	JHLB2469	proband	*MITF*: c.[332C > T];[=]	p.[A111V];[=]	exon 3	sporadic	normal	severe	+	+	−	Chen (2010)
	JHLB5132	proband	*SOX10*: c.(38369847_38379751_?)del	CNV		AD	profound	profound	+	+	−	this study
		grandfather	*SOX10*: c.(38369847_38379751_?)del	CNV			NA	NA	NA	+	−	
		mother	*SOX10*: c.(38369847_38379751_?)del	CNV			NA	NA	NA	NA		
		father (U)	*SOX10*: c.[=];[=]				NA	NA	NA	−	NA	
		brother (U)	*SOX10*: c.[=];[=]				normal	normal	NA	−	NA	
		grandmother (U)	*SOX10*: c.[=];[=]				NA	NA	NA	−	NA	
WS2	JHLB2091	proband	*MITF*: c.[326dupC];[=]	p.[S109fs];[=]	exon 3	AD	profound	(COR)	−	+	−	this study
		brother	*MITF*: c.[326dupC];[=]	p.[S109fs];[=]	exon 3		profound	(COR)	−	+	−	
		mother	*MITF*: c.[326dupC];[=]	p.[S109fs];[=]	exon 3		profound	profound	−	−	HD, FR	
		father (U)	*MITF*: c.[=];[=]	p.[=];[=]			profound	profound	−	−	NA	
	JHLB1623	proband	*MITF*: c.[389_399del];[=]	p.[Y130fs];[=]	exon 4	AD	profound	profound	−	+	FR	this study
		father	*MITF*: c.[389_399del];[=]	p.[Y130fs];[=]	exon 4		profound	normal	−	−	FR	
		brother	*MITF*: c.[389_399del];[=]	p.[Y130fs];[=]	exon 4		severe	severe	−	−	−	
		mother (U)	*MITF*: c.[=];[=]	p.[=];[=]			normal	normal	−	−	−	
	JHLB1593	proband	*MITF*: c.[550G > T];[=]	p.[E184X];[=]	exon 5	AD	severe	severe	−	+	−	this study
		mother	*MITF*: c.[550G > T];[=]	p.[E184X];[=]	exon 5		profound	profound	−	+	−	
	JHLB3463	proband	*MITF*: c.[796G > T];[=]	p.[E266X];[=]	exon 8	AD	profound	profound	−	+	−	this study
		mother	*MITF*: c.[796G > T];[=]	p.[E266X];[=]	exon 8		normal	profound	−	+	−	
	JHLB175	proband	*SOX10*: c.[400_417del];[=]	p.[L134fs];[=]	exon 2	sporadic (*de novo*)	profound	profound	−	+	HD, MA	this study
		father (U)	*SOX10*: c.[=];[=]	p.[=];[=]			normal	normal	−	−	NA	
		mother (U)	*SOX10*: c.[=];[=]	p.[=];[=]			normal	normal	−	−	NA	
	JHLB1632	proband	*SOX10*: c.[426G > C];[=]	p.[W142C];[=]	exon 2	sporadic	profound	profound	−	+	−	this study
		mother (U)	*SOX10*: c.[=];[=]	p.[=];[=]			NA	NA	−	−	NA	
	JHLB4310	proband	*SOX10*: c.[1195C > T];[=]	p.[Q399X];[=]	exon 4	sporadic (*de novo*)	profound	profound	−	−	MA, PT, MR	zazo seco (2017)
		father (U)	*SOX10*: c.[=];[=]	p.[=];[=]			NA	NA	NA	NA	NA	
		mother (U)	*SOX10*: c.[=];[=]	p.[=];[=]			NA	NA	NA	NA	NA	
	JHLB177	proband	*SOX10*: c.(?_38369393_38379751_?)del	CNV		sporadic (*de novo*)	profound	profound	−	+	MA	this study
		father (U)	*SOX10*: c.[=];[=]				NA	NA	−	−	−	
		mother (U)	*SOX10*: c.[=];[=]				NA	NA	−	−	−	
	JHLB3086	proband	*SOX10*: c.(?_38369393_38379751_?)del	CNV		sporadic (*de novo*)	moderate	(COR)	−	+	SD, MA	this study
		father (U)	*SOX10*: c.[=];[=]				NA	NA	−	−	NA	
		mother (U)	*SOX10*: c.[=];[=]				NA	NA	−	−	NA	
	JHLB2550	proband	*EDNRB*: c.[223delG];[=]	p.[D75fs];[=]		AD	moderate	severe	−	+	−	this study
		mother	*EDNRB*: c.[223delG];[=]	p.[D75fs];[=]			unilateral	NA	−	+	−	
Unclassifiable (WS1 or WS2)	JHLB3591	proband	*PAX3*: c.[318delC];[=]	p.[P106fs];[=]	exon 2	sporadic	profound	profound	NA	+	−	this study
	JHLB2343	proband	*PAX3*: c.[812G > A];[=]	p.[R271H];[=]	exon 6	AD	severe	(COR)	NA	+	−	Tassabehji (1995)
		father	*PAX3*: c.[812G > A];[=]	p.[R271H];[=]	exon 6		normal	normal	NA	+	−	
		mother (U)	*PAX3*: c.[=];[=]	p.[=];[=]			NA	NA	−	−	NA	
WS4	JHLB4270	proband	*SOX1*0: c.[781_793del];[=]	p.[R261fs];[=]	exon 4	sporadic	severe	severe	−	+	HI, PT	this study
	JHLB3480	proband	*SOX10*: c.[859delT];[=]	p.[S287fs];[=]	exon 4	sporadic (*de novo*)	severe	severe	−	+	HI, MA, AS	this study
		father (U)	*SOX10*: c.[=];[=]	p.[=];[=]			normal	normal	−	−	NA	
		mother (U)	*SOX10*: c.[=];[=]	p.[=];[=]			normal	normal	−	−	NA	
		sister (U)	*SOX10*: c.[=];[=]	p.[=];[=]			normal	normal	−	−	NA	
		sister (U)	*SOX10*: c.[=];[=]	p.[=];[=]			normal	normal	−	−	NA	

U: Unaffected family member, AD: Autosomal dominant, CNV: Copy number variation, COR: Conditioned orientation audiometory.

HD: Hair discoloration, SD: Skin discoloration, FR: Freckles, HI: Hirschsprung disease, MA: Malformation of inner ear, PT: Ptosis, MR: Mental retardation, AS: Asperger syndrome.

The reference cDNA sequences NM_181457 for *PAX3*, NM_000248 for *MITF*, NM_006941 for *SOX10*, NM_000115 for *EDNRB*.

### Mutation spectrum and clinical features of other syndromic hearing loss patients

We also conducted genetic analysis of other syndromic hearing loss patients (five osteogenesis imperfecta cases, one spondyloepiphyseal dysplasia congenita case, three Stickler syndrome cases, three CHARGE syndrome cases, one Jervell and Lange-Nielsen syndrome case, one auditory neuropathy with optic atrophy case, and 36 Pendred syndrome cases). The diagnostic rate for each syndrome was 60% for osteogenesis imperfecta with *COL1A1* variants (3/5), 100% for spondyloepiphyseal dysplasia congenita with a *COL2A1* variant (1/1), 100% for Stickler syndrome with *COL11A1*, *COL11A2* variants (2/3, 1/3), 33% for CHARGE syndrome with a *CHD7* variant (1/3), 100% for Jervell and Lange-Nielsen syndrome with a *KCNQ1* variant (1/1), 100% for auditory neuropathy with a *OPA1* mutation (1/1), and 89% for Pendred syndrome with *SLC26A4* variants (32/36). Tables 5, 6 provide summaries of the identified variants and clinical features of the probands and all family members harboring the same variants (the pedigrees and audiograms of these cases are shown in Supplementary Figs S3, S4). The identified variants in all three probands with osteogenesis imperfecta were previously reported truncating variants. All four affected cases had easily fractured bones, blue sclera and hearing loss. The severity of hearing loss varied from mild to severe with air-bone gap. All three probands were from autosomal dominant families.

**Table 5:** Genetic diagnosis results and clinical features of osteogenesis imperfecta, spondyloepiphyseal dysplasia congenita, Stickler syndrome, Jervell Lange-Nielsen syndrome and auditory neuropathy with optic atrophy patients and family members.

Proband	Family	Nucleotide change	Amino Acid change	Location	Hereditary form	Severity of hering loss		Other Clinical Features	Reference
						Rt.ear	Lt.ear		
**Osteogenesis imperfecta**									
JHLB459	proband	*COL1A1*:c.[903 + 1 G > A];[=]		intron 14	AD	moderate	mild	easy fracture、blue sclera	Schleit (2015)
JHLB-3127	proband	*COL1A1*:c.[1414 C > T];[=]	p[R472X];[=]	exon 21	AD	profound	profound	easy fracture、blue sclera	Pollitt (2006)
	mother	*COL1A1*:c.[1414 C > T];[=]	p[R472X];[=]	exon 21		severe	profound	easy fracture、blue sclera	
	father	*COL1A1*:c.[=];[=]	p[=];[=]			normal	normal	—	
JHLB325	proband	*COL1A1*:c.[2127 + 2 T > A];[=]		intron 31	AD	normal	moderate	blue sclera, otosclerosis, easy fracture	Shaheen (2012)
**Spondyloepiphyseal dysplasia congenita**									
JHLB1192	proband	*COL2A1*:c.[3198_3206del];[=]	p.[1066_1069del.];[=]	exon 46	AD	profound	profound	cleft palate, short stature, short extremities	this study
	father	*COL2A1*:c.[3198_3206del];[=]	p.[1066_1069del.];[=]	exon 46		moderate	severe	cleft palate, short stature, short extremities	
	mother	*COL2A1*:c.[=];[=]	p[=];[=]			NA	NA		
**Stickler syndrome**									
JHLB4194	proband	*COL11A1*:c.[1737 + 2 T > C];[=]		intron 17	AD	mild	mild	cleft palate,myopia (congenital)	this study
	mother	*COL11A1*:c.[1737 + 2 T > C];[=]		intron 17		mild	mild	cleft palate,myopia (congenital,mild)	
	father (U)	*COL11A1*:c.[=];[=]				normal	normal		
JHLB4190	proband	*COL11A1*:c.[3117_3152del];[=]	p.[1039_1051del];[=]	exon 41	spoadic(*de novo*)	mild	mild	cleft palate,myopia (congenital)	this study
	father (U)	*COL11A1*:c.[=];[=]				normal	normal		
	mother (U)	*COL11A1*:c.[=];[=]				normal	normal		
	brother (U)	*COL11A1*:c.[=];[=]				normal	normal		
JHLB4181	proband	*COL11A2*:c.[4392 + 1 G > A];[=]		intron 61	AD	mild	mild	uvula bifida,myopia (acquired,mild)	Vikkula (1995)
	daughter	*COL11A2*:c.[4392 + 1 G > A];[=]		intron 61		normal	normal	cleft palate	
	Son (U)	*COL11A2*:c.[4392 + 1 G > A];[=]		intron 61		normal	normal		
	brother	*COL11A2*:c.[4392 + 1 G > A];[=]		intron 61		moderate	mild	cleft palate	
	mother	*COL11A2*:c.[4392 + 1G > A];[=]		intron 61		moderate	moderate		
**CHARGE syndrome**									
#JHLB448	proband	*CHD7:*c.[808delG];[=]	p.[A270fs];[=]	exon 2	spoadic	profound	profound	cardiac malformation, laryngomalacia, lower cranial nerve disorder, coloboma	Sanlaville (2006)
	mother (U)	*CHD7:*c.[=];[=]	p[=];[=]			normal	normal		
**Jervell and Lange**-**Nielsen syndrome**									
JHLB4860	proband	*KCNQ1*: c.[1484_1485del];[520 C > T]	p.[T495fs];[R174C]	exon 11.exon 3	AR	moderate	(COR)	bilateral superior canal dehiscence	Napolitano (2005),Donger (1997)
	father	*KCNQ1*: c.[520 C > T];[=]	p.[R174C];[=]	exon 3		normal	normal		
	mother	*KCNQ1*: c.[1484_1485del];[=]	p.[T495fs];[=]	exon 11		normal	normal		
**Auditory neuropathy with optic atrophy**									
JHLB-2582	proband	*OPA1*: c.[892 A > C];[=]	p.[S298R];[=]	exon 9	AD	moderate	moderate	amblyopia childhood onset. optic nerve atrophy	this study

U: Unaffected family member, AD: Autosomal dominant, AR: Autosomal recessive, COR: Conditioned orientation response audiometory.

The reference cDNA sequences NM_000088 for *COL1A1*, NM_001844 for *COL2A1*, NM_001854 for *COL11A1*, NM_080680 for *COL11A2*, NM_017780 for *CHD7*, NM_000218 for *KCNQ1*, NM_015560 for *OPA1*.

**Table 6:** Genetic diagnosis results and clinical features of Pendred syndrome patients.

Proband	Nucleotide change	Amino Acid change	Severity of hearing loss		Malformation of inner ear	Goiter
			Rt.(dB)	Lt.(dB)		
#752	c.[919-2 A > G];[1652insT]	c.[919-2 A > G];[1652insT]	101.25	103.75	EVA	+
#1045	c.[2168 A > G];[2168 A > G]	p[.H723R];[H723R]	90	98.75	EVA, IP2	+
#2010	c.[2168 A > G];[601-1 G > A]	p[.H723R];c.[601-1 G > A]	77.5	96.25	EVA	+
#2331	c.[2168 A > G];[2168 A > G]	p[.H723R];[H723R]	92.5	102.5	EVA	+
#2538	c.[2168 A > G];[2168 A > G]	p[.H723R];[H723R]	102.5	57.5	EVA	+
#2798	c.[2168 A > G];[2168 A > G]	p[.H723R];[H723R]	56.25	98.75	EVA	+
#3074	c.[2168 A > G];[1707 + 5 G > A]	p.[H723R];c.[1707 + 5 G > A]	107.5	107.5	EVA	+
#3994	c.[2168 A > G];[601-1 G > A]	p[.H723R];c.[601-1 G > A]	NA	NA	EVA	+
#4386	c.[2168 A > G];[2168 A > G]	p[.H723R];[H723R]	83.75	92.5	EVA	+
#4486	c.[1707+5 G > A];[1707 + 5 G > A]	c.[1707 + 5 G > A];c.[1707 + 5 G > A]	72.5	98.75	EVA	+
#4490	c.[1229 C > T];[1229 C > T]	p.[T410M];[T410M]	92.5	97.5	EVA	+
#4545	c.[2168 A > G];[1707 + 5 G > A]	p.[H723R];c.[1707 + 5 G > A]	95	33.75	EVA	+
JHLB40	c.[2168 A > G];[1707 + 5 G > A]	p.[H723R];c.[1707 + 5 G > A]	78.75	76.25	EVA	+
JHLB401	c.[2168 A > G];0.1707 + 5 G > A	p.[H723R];c.[1707 + 5 G > A]	115	107.5	EVA	+
JHLB427	c.[1229 C > T];[1229 C > T]	p.[T410M];[T410M]	97.5	93.75	EVA	+
JHLB507	c.[2168 A > G];[1229 C > T]	p.[H723R];[T410M]	80	62.5	EVA	+
JHLB572	c.[2168 A > G];[1229 C > T]	p.[H723R];[T410M]	108.25	111.25	EVA	+
JHLB575	c.[1579 A > C];[1707 + 5 G > A]	p.[T527P];c.[1707 + 5 G > A]	110	77.5	EVA	+
JHLB915	c.[2168 A > G];[367 C > T]	p.[H723R];[P123S]	115	115	EVA	+
JHLB1392	c.[2168 A > G];[601-1 G > A]	p.[H723R];c.[601-1 G > A]	111.25	100	EVA	+
JHLB1790	c.[2168 A > G];[147 C > G]	p.[H723R];[S49R]	82.5	93.75	EVA	+
JHLB2150	c.[2168 A > G];[919-2 A > G]	p.[H723R];c.[919-2 A > G]	105	91.25	EVA	+
JHLB2286	c.[2168 A > G];[919-2 A > G]	p.[H723R];c.[919-2 A > G]	108.75	112.5	EVA	+
JHLB2485	c.[1579 A > C];[1229 C > T]	p.[T527P];p.[T410M]	NA	NA	EVA	+
JHLB2571	c.[2168 A > G];[919-2 A > G]	p.[H723R];c.[919-2 A > G]	100	115	EVA	+
JHLB2849	c.[2168 A > G];[1001 + 1 G > A]	p.[H723R];c.[1001 + 1 G > A]	97.5	52.5	EVA	+
JHLB2857	c.[2168 A > G];[919-2 A > G]	p.[H723R];c.[919-2 A > G]	107.5	113.75	EVA	+
JHLB3229	c.[2168 A > G];[1652insT]	p.[H723R];c.[1652insT]	102.5	58.75	EVA	+
JHLB3735	c.[1343 C > T];[1229 C > T]	p.[S448L];[T410M]	53.75	58.75	EVA, IP2	+
JHLB4048	c.[2168 A > G];[1229 C > T]	p.[H723R];[T410M]	96.25	105	EVA, IP2	+
JHLB4679	c.[2168 A > G];[1648insT]	p.[H723R];c.[1648insT]	78.75	67.5	EVA	+
JHLB4876	c.[1174 A > T];[2162 C > T]	p.[N392Y];[T721M]	105	105	EVA	+

The reference cDNA sequence NM_000441 for *SLC26A4*.

The proband with spondyloepiphyseal dysplasia congenita had a novel truncating variant in *COL2A1*. She and her father, who harbored the same variant, had characteristic clinical features (cleft palate, short stature and short extremities). Their hearing level was severe to profound sensorineural hearing loss.

With regard to Stickler syndrome, we identified pathogenic variants in the *COL11A1* (two cases) and *COL11A2* (one case) genes. All identified variants were truncating (two were splice site, one was frameshift), with the two variants in *COL11A1* being novel. One *COL11A1* and one *COL11A2* variant were identified from an autosomal dominant family, and one *COL11A1* variant was identified from a sporadic case (*de novo*). As to the clinical features of the probands and all family members harboring the same causative gene variants (8 patients in total), hearing loss was observed in 75% of cases (3/3 with *COL11A1* variants, 3/5 with *COL11A2* variants), with the severity of hearing loss being mild to moderate. Two children of the proband with a *COL11A2* variant (JHLB4181) carried the same variant but had normal hearing. Seventy-five percent of cases (6/8) had a cleft palate or uvula bifida (3/3 with *COL11A1* variants, 3/5 with *COL11A2* variants), and all three cases with *COL11A1* variants had congenital myopia. One case harboring a *COL11A2* variant, who was the son of the proband, had no symptoms.

A novel *OPA1* variant was identified in one case who suffered auditory neuropathy with optic atrophy. Two other pathogenic amino acid substitutions have been previously identified in the same position. The proband had amblyopia since infancy, and bilateral moderate sensorineural hearing loss. OAE (Otoacoustic emission) presented a normal response, the ABR (Auditory Brainstem Response) threshold was out of scale, and MRI (magnetic resonance imaging) showed bilateral cochlear nerve hypoplasia. The proband’s mother had similar symptoms (no DNA sample was available).

With regard to Pendred syndrome, we identified *SLC26A4* variants in 32 probands with autosomal recessive inheritance or sporadic cases. No variants in *KCNJ10* and *FOXI1* were identified in cases with heterozygous *SLC26A4* variants.

## Discussion

In this study, we conducted a comprehensive analysis of Japanese syndromic hearing loss patients to clarify mutation spectrums and clinical features by using NGS analysis with a multiple syndromic targeted resequencing panel. This analysis had a high diagnostic rate (56%) and was suitable for comprehensive analysis. Further, it allowed us to clarify the types and frequency of causative genes in Japanese syndromic hearing loss patients. In addition, it was particularly useful in cases that only partially fulfilled the respective diagnostic criteria. To the best of our knowledge, this is the first study using targeted resequencing panel analysis for multiple syndromic hearing loss patients.

With regard to BO/BOR syndrome, the causative variants were identified in 32% (19/59) of probands (16 typical, 43 atypical). The diagnostic rate was increased to 75% when we restricted the analysis to typical BO/BOR cases (12/16). Krug *et al*. reported the results of genetic analysis for a large number of BO/BOR patients and identified the causative variants in 36% of cases. Similar to this study, the diagnostic rate was increased to 76% when they restricted subjects to typical BO/BOR cases^28^. Unzaki *et al*. analyzed 36 Japanese families with clinically diagnosed BO/BOR syndrome and identified causative genes in 72% of them^29^. Thus, the diagnostic rate in this study was similar to the rates in these previous reports. *EYA1* variants account for 95% of the causative gene variants identified in this study. Similarly, *EYA1* was commonly identified in BO/BOR cases in previous studies; 85% in Japanese patients^29^ and 93% in French patients^28^. *SIX1* variants were identified in 5% (1/19) of the genetically diagnosed cases in this study. This percentage was similar to the results of previous reports^28,29^. No causative gene variants were identified in 25% of the typical BO/BOR syndrome cases in this study. There is a possibility that variants in other genes (such as *SALL1*) or genomic rearrangement (inversion or translocation in chromosome 8) may contribute to these cases. In this study, we also identified one copy number loss with a 2.8 Mb deletion of 8q13.2-q13.3 including the *EYA1* gene in one familial case. The frequency of one copy number loss of the *EYA1* gene was 6% (1/18) in this study. In other reports, copy number loss of the *EYA1* gene was also involved in BO/BOR syndrome, with 7% to 10% or more of cases caused by *EYA1* copy number loss^28,29^. The most frequent clinical feature was hearing loss, which was observed in 97% of cases (31/32), followed by preauricular pits in 88% (29/33). In other reports, the most frequent clinical feature was also hearing loss; however, the frequencies of other symptoms varied, with the frequency of renal symptoms higher in some reports^28–30^. Chen *et al*. reported renal anomalies in 67% of affected individuals^31^, with about 6% of them progressing to renal failure^32^. Some of them were asymptomatic in the first decade but required dialysis or renal transplantation in adulthood^33,34^. In this study, only one case showed congenital renal anomalies. One plausible reason for this lower rate of renal anomalies was that we enrolled BO/BOR candidate patients, and information regarding renal anormalies was available for only a limited number of patients (renal ultrasonographic information was available for only 7/34 cases). Therefore, more cases may have had renal symptoms. In cases in which BO/BOR syndrome is suspected clinically or genetically, even in the absence of renal dysfunction in early childhood, renal examination may be important.

It is noteworthy that three cases from two unrelated families with *EYA1* variants presented visual symptoms (progressive disturbance of vision, amblyopia and hypermetropia), but visual symptoms are not typically associated with BOR syndrome. *EYA1* is needed for the formation of the anterior portion of the eye^35^. Azuma *et al*. reported one case who presented with congenital cataracts with a BOR phenotype (cervical fistula, unilateral multicystic kidney and conductive hearing loss due to ossicular malformations), and others have also reported cases with visual symptoms (dysopia, cataract, micrognathia, and iris coloboma)^28,29,36–38^. The frequency of amblyopia is reported to be 3.0% to 3.2% in the general population^39,40^, but the frequency of visual symptoms in the *EYA1*-related BO/BOR patients in this study was a little higher (9%). There is a possibility that visual symptoms actually represent a rare clinical feature of BO/BOR syndrome.

Waardenburg syndrome was subdivided into four types based on the clinical findings, and each causative gene was identified. We successfully identified the genetic causes in 80% of WS1 probands (4/5), 71% of WS2 probands (10/14), and 100% of WS4 probands (2/2). Hoth *et al*. reported that point mutations in *PAX3* have been identified in more than 90% of affected individuals with WS1 or WS3^41,42^. In this study, we identified one case each with *MITF* and *SOX10* variants from WS1. *MITF* and *SOX10* variants were generally identified from WS2 or WS4 patients. Similarly, *MITF*, *EDNRB*, and *SOX10* variants were identified from WS1 patients in previous reports^42–44^. The cause of this inconsistency between phenotype and genotype may be 1) a new genotype-phenotype correlation or 2) the wider distance between the inner canthus in the Japanese population. In the Japanese literature, Motomura reported the inter-inner canthal, inter-outer canthal and inter-pupillary distance for each age group among Japanese (published in Japanese)^45^. It appears that the W-index calculated from these data may exceed 1.95 in many age groups (Supplementary Fig. S5). In future, it may be necessary to consider ethnic differences when evaluating the W-index. Among the WS2 cases, we identified the causative variants in 29% of cases with *MITF*, in 36% with *SOX10*, and in 7% with *EDNRB*. Pingault *et al*. reported that *MITF* mutations were involved in about 15% of cases, 15% with *SOX10,* and *EDNRB* and *SNAI2* are a small percentage among WS2 patients^46^. Bocángel *et al*. reported that *MITF* variants and *SOX10* variants were observed in 12% and 20% of South-eastern Brazilian WS2 cases, respectively^47^. Sun *et al*. also reported that the rates of causative genes observed in Chinese WS2 cases were 34% for *MITF* and 45% for *SOX10*, respectively^48^. Taken together, these results indicate that *SOX10* variants may be more frequently identified in East Asian WS2 cases.

It is worth noting that we also identified one copy number loss of the *SOX10* gene using NGS read depth data and confirmed by aCGH in three cases. Two probands had a large deletion within the chromosome 22q13.1, a proband had the whole *SOX10* gene deletion, and the other proband in a familial case had a partial deletion of *SOX10*. To date, more than 20 cases caused by copy number variation in *PAX3* or *SOX10* have been reported^42,47,49–54^. We identified one *SOX10*-assiociated WS case with developmental delay and one with Asperger syndrome. Both of these cases carried truncation variants; however, no cases were observed with developmental delay among the *SOX10* CNV cases. Thus, the association between genotype and developmental delay phenotype remains unclear.

In addition, we also identified one familial WS case with variations in phenotype among family members. In the *MITF* family (JHLB1623), the father had only unilateral hearing loss and excessive freckles, and her younger brother had only bilateral severe sensorineural hearing loss, but both had the same variant. It is usually difficult to suspect WS from clinical findings and family history; therefore, the comprehensive syndromic hearing loss panel was useful in such cases who only partially fulfilled the diagnostic criteria.

In conclusion, this analysis using NGS with a multiple syndromic targeted resequencing panel was useful for identifying the causative genes in multiple syndromic hearing loss patients in a short time and with a high diagnostic rate.

## Subjects and Methods

### Subjects

In this study we enrolled total 140 probands with possible syndromic hearing loss who carried hearing loss with one or more associated symptoms typical of each syndrome from our hearing loss cohort of 5,137 patients gathered from 67 cooperative research institutes in Japan as described elsewhere^55^ (Detailed numbers for each syndrome are listed in Table 2). We also collected data on the hearing level of each proband and their family members. The severity of hearing was classified as mild (20–40 dB), moderate (41–70 dB), severe (71–95 dB), or profound (>95 dB). With regard to BO/BOR syndrome, we enrolled the patients who fulfilled the criteria (typical and atypical) described previously^4^. Regarding auditory neuropathy, the probands with pathogenic variants in *OTOF* and *DFNB59* were excluded from this study.

Written informed consent was obtained from all patients or their guardians. This study was approved by the Shinshu University Ethical Committee as well as the respective Ethical Committees of the other participating institutions listed below. Akita University Ethical Committee, Iwate Medical University Ethical Committee, Tohoku Rosai Hospital Ethical Committee, Fukushima Medical University Ethical Committee, Yamagata University Ethical Committee, Dokkyo Medical University Ethical Committee, TAKASAKI Ear Nose & Throat Clinic Ethical Committee, Niigata University Ethical Committee, Tokyo Medical University Ethical Committee, Jikei University Ethical Committee, Toranomon Hospital Ethical Committee, Kitasato University Ethical Committee, International University of Health and Welfare Mita Hospital Ethical Committee, National Rehabilitation Center for Persons with Disabilities Ethical Committee, Keio University Ethical Committee, Hamamatsu University Ethical Committee, Shiga University Ethical Committee, Shiga Medical Center for Children Ethical Committee, Osaka University Ethical Committee, Kobe City Medical Center General Hospital Ethical Committee, Hyogo College of Medicine Ethical Committee, Kyoto Prefectural University Ethical Committee, Okayama University Ethical Committee, Yamaguchi University Ethical Committee, Ehime University Ethical Committee, Kyushu University Ethical Committee, Kanda ENT Clinic Ethical Committee, Nagasaki University Ethical Committee, Miyazaki University Ethical Committee, Kagoshima University Ethical Committee, Ryukyus University Ethical Committee, Sapporo Medical University Ethical Committee, Tohoku University Ethical Committee, Jichi Medical University Ethical Committee, Gunma University Ethical Committee, Jyuntendo University Ethical Committee, Yokohama City University Ethical Committee, Mejiro University Ethical Committee, Saitama Medical University Ethical Committee, Abe ENT Clinic Ethical Committee, Tokyo Medical Center Institute of Sensory Organs Ethical Committee, Jichi University Saitama Medical Center Ethical Committee, Aichi Children’s Health Medical Center Ethical Committee, Chubu Rosai Hospital Ethical Committee, Kyoto University Ethical Committee, Mie University Ethical Committee, Kansai Medical University Ethical Committee, Kobe University Ethical Committee, Osaka Medical Center and Research Institute for Maternal and Children Health Ethical Committee, Wakayama Medical University Ethical Committee, Kouchi University Ethical Committee, Hiroshima University Ethical Committee, Hiroshima City Hiroshima Citizen Hospital Ethical Committee, Fukuoka University Ethical Committee, Kurume University Ethical Committee, National Defense Medical College Ethical Committee, Tokai University Ethical Committee, Hokkaido University Ethical Committee, Kanagawa Children’s Medical Center Ethical Committee, Tokyo Medical and Dental University Ethical Committee, Hirosaki University Ethical Committee, Tokyo Metropolitan Children’s Medical Center Ethical Committee, Hakodate Central General Hospital Ethical Committee, Osaka Red Cross Hospital Ethical Committee, Hiroshima Prefectural Hospital Ethical Committee, Nara Medical University Ethical Committee, Tsukuba University Ethical Committee. All methods were performed in accordance with the Guidelines for Genetic Tests and Diagnoses in Medical Practice of the Japanese Association of Medical Sciences and the Declaration of Helsinki as required by Shinshu University.

## Methods

### Amplicon Library Preparation

An Amplicon library was prepared with an Ion AmpliSeq^TM^ Custom Panel (Applied Biosystems, Life Technologies) for 36 target genes reported to cause syndromic hearing loss. We selected the 36 genes associated with 14 types of syndromic hearing loss commonly observed in practical settings. We also referred to the hereditary hearing loss homepage (https://hereditaryhearingloss.org) to select these genes. The responsible genes for Usher syndrome were not included in our syndromic hearing loss targeting panel as these genes were included in the non-syndromic hearing loss panel reported in a previous paper^55^. To avoid any overlap between these two panels, we removed the genes associated with Usher syndrome from our panel. The panel contained the following genes: *EYA1*-*SIX1*-*SIX5 for* BOR syndrome; *PAX3*-*MITF*-*SNAI2*-*EDNRB*-*EDN3*-*SOX10* for Waardenburg syndrome; *COL2A1*-*COL11A1*-*COL11A2*-*COL9A1*-*COL9A2*-*COL9A3*-*COL1A1*-*COL4A3*-*COL4A4*-*COL4A5* for connective tissue disorder including osteogenesis imperfecta, spondyloepiphyseal dysplasia congenita, Stickler syndrome, and Alport syndrome; *CHD7*-*SEMA3E* for CHRGE syndrome; *SLC26A4*- *FOXI1*-*KCNJ10* for Pendred syndrome; *KCNQ1*-*KCNE1* for Jervell Lange-Nielsen syndrome; *NDP* for Norrie disease; *TCOF1*-*POLR1C* for Treacher-Collins syndrome, *HSD17B4*-*HARS2*-*CLPP*-*LARS2* for Perrault syndrome; *OPA1* for auditory neuropathy with optic atrophy and *GDF6*-*MEOX1* for Klippel Feil syndrome.

### Emulsion PCR and sequencing

The emulsion PCR and NGS (next-generation sequencing) were performed with an Ion Proton system using the Ion Proton 200 sequencing Kit and an Ion P1 Chip (ThermoFisher Scientific, Waltham, MA, USA) according to the manufacturer’s instructions.

### Base call and data analysis

The sequence data were mapped against the human genome sequence (build GRCh37/hg19) with the Torrent Mapping Alignment Program. After sequencing mapping, the DNA variant regions were piled up with Torrent Variant Caller plug-in software. After variant detection, variant effects were analyzed using the ANNOVAR software^56,57^.

### Direct sequencing

After the filtering process, described previously^55^, we performed confirmation of the identified variant and family segregation analysis by Sanger sequencing.

### CNV (Copy Number Variation) analysis

CNV analysis was performed with NGS analysis read depth data according to the method described in a previous report^58^.

### aCGH (Array Comparative Genomic Hybridization)

To confirm the CNVs identified from NGS read depth data, we performed array CGH analysis with the Agilent 8 × 60 K whole genome array (Agilent Technologies, Santa Clara, CA). We used the same DNA samples as for the amplicon re-sequencing, and quality assessment was also carried out. Ten microliters of genomic DNA solution (0.5ug of DNA) were fragmented, labeled with cyanine-3 for reference DNA samples and cyanine-5 for subjects, and then hybridized. Scanning of the array was carried out according to the manufacturer’s recommended protocols. Scanned aCGH data were analyzed using CytoGenomics software version 3.0.6.6 (Agilent Technologies).

## Supplementary information

Table S1, Table S2, Figure S1, Figure S2, Figure S3, Figure S4, Figure S5

## References

[1] Morton CC, Nance WE (2006). Newborn hearing screening-a silent revolution. N. Engl. J. Med..

[2] Alford RL (2014). American college of medical genetics and genomics guideline for the clinical evaluation and etiologic diagnosis of hearing loss. Genet. Med..

[3] Fraser FC, Sproule JR, Halal F (1980). Frequency of the branchio-oto-renal (BOR) syndrome in children with profound hearing loss. Am. J. Med. Genet..

[4] Chang EH (2004). Branchio-oto-renal syndrome: the mutation spectrum in *EYA1* and its phenotypic consequences. Hum. Mutat..

[5] Waardenburg PJ (1951). A new syndrome combining developmental anomalies of the eyelids, eye brows, and nose root with pigmentary defects of the iris and head hair and with congenital deafness. Am. J. Hum. Genet..

[6] Read AP, Newton VE (1997). Waardenburg syndrome. J. Med. Genet..

[7] Arias S (1971). Genetic heterogeneity in the Waardenburg syndrome. Birth Defects Orig. Artic. Ser..

[8] Klein D (1983). Historical background and evidence for dominant inheritance of the Klein-Waardenburg syndrome (type III). Am. J. Med. Genet..

[9] Fraser, G. R. The causes of profound deafness in childhood. *Baltimore: Johns Hopkins university press* (1976).

[10] Sillence DO, Senn A, Danks DM (1979). Genetic heterogeneity in osteogenesis imperfecta. J. Med. Genet..

[11] Anderson IJ, Goldberg RB, Marion RW, Upholt WB, Tsipouras P (1990). Spondyloepiphyseal dysplasia congenita: genetic linkage to type II collagen (*COL2A1*). Am. J. Hum. Genet..

[12] Stickler GB (1965). Hereditary progressive arthro-ophthalmopathy. Mayo Clinic Proc..

[13] Ahmad NN (1991). Stop codon in the procollagen II gene (*COL2A1*) in a family with the Stickler syndrome (arthro-ophthalmopathy). Proc. Natl. Acad. Sci. USA.

[14] Richards AJ (1996). A family with Stickler type 2 has a mutation in the *COL11A1* gene resulting in the substitution of glycine 97 by valine in alpha 1(IX) collagen. Hum. Mol. Genet..

[15] Van Camp G (2006). A new autosomal recessive form of Stickler syndrome is caused by a mutation in the *COL9A1* gene. Am. J. Hum. Genet..

[16] Baker S (2011). A loss of function mutation in the *COL9A2* gene causes autosomal recessive Stickler syndrome. Am. J. Med. Genet. A..

[17] Vikkula M (1995). Autosomal dominant and recessive osteochondrodysplasias associated with the *COL11A2* locus. Cell.

[18] Pirson Y (1999). Making the diagnosis of Alport’s syndrome. Kidney Int..

[19] Longo I (2002). *COL4A3*/*COL4A4* mutation: from familial hematuria to autosomal-dominant or recessive Alport syndrome. Kidney Int..

[20] Marcocci E (2009). Autosomal dominant Alport syndrome: molecular analysis of the *COL4A4* gene and clinical outcome. Nephrol. Dial. Transplant..

[21] Hall BD (1979). Choanal atresia and associated multiple anomalies. J. Pediatr..

[22] Hittner HM, Hirsch NJ, Kreh GM, Rudolph AJ (1979). Colobomatous micriphthalmia, heart disease, hearing loss, and mental retardation–a syndrome. J. Pediatr. Ophthalmol. Strabismus.

[23] Pagon RA, Graham JM, Zonana J, Yong SL (1981). Coloboma, congenital heart disease, and choanal atresia with multiple anomalies: CHARGE association. J. Pediatr..

[24] Lalani SR (2004). *SEMA3E* mutation in a patient with CHARGE syndrome. J. Med. Genet..

[25] Neyroud N (1997). A novel mutation in the potassium channel gene *KVLQT1* causes the Jervell and Lange-Nielsen cardioauditory syndrome. Nat. Genet..

[26] Schulze-Bahr E (1997). *KCNE1* mutations cause Jervell and Lange-Nielsen syndrome. Nat. Genet..

[27] Everett LA (1997). Pendred syndrome is caused by mutations in a putative sulphate transporter gene (PDS). Nat. Genet..

[28] Krug P (2011). Mutation screening of the *EYA1*, *SIX1*, and *SIX5* genes in a large cohort of patients harboring branchio-oto-renal syndrome calls into question the pathogenic role of *SIX5* mutations. Hum. Mutat..

[29] Unzaki A (2018). Clinically diverse phenotypes and genotypes of patients with branchio-oto-renal syndrome. J. Hum. Genet..

[30] Morisada N, Nozu K, Iijima K (2014). Branchio-oto-renal syndrome: Comprehensive review based on nationwide surveillance in Japan. Pediatr. Int..

[31] Chen A (1995). Phenotypic manifestations of branchio-oto-renal syndrome. Am. J. Med. Genet..

[32] Misra M, Nolph KD (1998). Renal failure and deafness: Branchio-oto-renal syndrome. Am. J. Kidney Dis..

[33] Annear NM, Gale DP, Loughlin S, Dorkins HR, Maxwell PH (2008). End-stage renal failure associated with congenital deafness. NDT Plus.

[34] Gigante M (2013). Branchio-oto-renal syndrome (BOR) associated with focal glomerulosclerosis in a patient with a novel *EYA1* splice site mutation. BMC Nephrol..

[35] Xu PX, Woo I, Her H, Beier DR, Maas RL (1997). Mouse eya homologues of the Drosophila eyes absent gene require Pax6 for expression in lens and nasal placode. Development.

[36] Azuma N, Hirakiyama A, Inoue T, Asaka A, Yamada M (2000). Mutations of a human homologue of the Drosophila eyes absent gene (*EYA1*) detected in patients with congenital cataracts and ocular anterior segment anomalies. Hum. Mol. Genet..

[37] Rickard S, Boxer M, Trompeter R, Bitner-Glindzicz M (2000). Importance of clinical evaluation and molecular testing in the branchio-oto-renal (BOR) syndrome and overlapping phenotypes. J. Med. Genet..

[38] Sanchez-Valle A (2010). HERV-mediated genomic rearrangement of *EYA1* in an individual with branchio-oto-renal syndrome. Am. J. Med. Genet. A.

[39] Anderson RL, Baumgartner SA (1980). Amblyopia in ptosis. Arch. Ophthalmol..

[40] Dray JP, Leibovitch I (2002). Congenital ptosis and amblyopia: a retrospective study of 130 cases. J. Pediatr. Ophthalmol. Strabismus.

[41] Hoth CF (1993). Mutations in the paired domain of the human *PAX3* gene cause Klein-Waardenburg syndrome (WS-III) as well as Waardenburg syndrome type I (WS-I). Am. J. Hum. Genet..

[42] Milunsky JM, Maher TA, Ito M, Milunsky A (2007). The value of MLPA in Waardenburg syndrome. Genet. Test..

[43] Morimoto N (2018). Homozygous *EDNRB* mutation in a patient with Waardenburg syndrome type1. Auris Nasus Larynx.

[44] Suzuki N (2018). A case report of reversible generalized seizures in a patient with Waardenburg syndrome associated with a novel nonsense mutation in the penultimate exon of *SOX10*. BMC Pediatr..

[45] Honmura Yoshio (1969). Clinical and Genetic Study of Waardenhurg-Klein Syndrome in Japanese. AUDIOLOGY JAPAN.

[46] Pingault V (2010). Review and update of mutations causing Waardenburg syndrome. Hum. Mutat..

[47] Bocángel MAP (2018). Waardenburg syndrome: Novel mutations in a large Brazilian sample. Eur. J. Med. Genet..

[48] Sun L (2016). Molecular etiology and genotype-phenotype correlation of Chinese Han deaf patients with type I and type II Waardenburg syndrome. Sci. Rep..

[49] Tassabehji M (1995). The mutational spectrum in Waardenburg syndrome. Hum. Mol. Genet..

[50] Bondurand N (2007). Deletions at the *SOX10* gene locus cause Waardenburg syndrome types 2 and 4. Am. J. Hum. Genet..

[51] Siomou E (2012). A 725 kb deletion at 22q13.1 chromosomal region including *SOX10* gene in a boy with a neurologic variant of Waardenburg syndrome type 2. Eur. J. Med. Genet..

[52] Jelena B, Christina L, Eric V, Fabiola QR (2014). Phenotypic variability in Waardenburg syndrome resulting from a 22q12.3-q13.1 microdeletion involving. SOX10. Am. J. Med. Genet. A.

[53] Wenzhi H (2015). Heterozygous deletion at the SOX10 gene locus in two patients from a Chinese family with Waardenburg syndrome type II. Int. J. Pediatr. Otorhinolaryngol..

[54] Li H (2017). Identification of a novel de novo heterozygous deletion in the SOX10 gene in Waardenburg syndrome type II using next-generation sequencing. Genet. Test. Mol. Biomarkers..

[55] Kitano T (2017). *POU4F3* mutation screening in Japanese hearing loss patients: Massively parallel DNA sequencing-based analysis identified novel variants associated with autosomal dominant hearing loss. PLos One.

[56] Wang K, Li M, Hakonarson H (2010). ANNOVAR: functional annotation of genetic variants from high-through-put sequencing data. Nucleic. Acids. Res..

[57] Chang X, Wang K (2012). wANNOVAR: annotating genetic variants for personal genomes via the web. J. Med. Genet..

[58] Nishio SY, Moteki H, Usami SI (2018). Simple and efficient germline copy number variant visualization method for the Ion Ampliseq^TM^ custom panel. Mol. Genet. Genomic Med..

[59] Tassabehji M (2008). Mutations in *GDF6* are associated with vertebral segmentation defects in Klippel-Feil syndrome. Hum. Mutat..

[60] Huang T, Santarelli R, Starr A (2009). Mutation of *OPA1* gene causes deafness by affecting function of auditory nerve terminals. Brain Res..

