# Supplementary material for: Comprehensive analysis of syndromic hearing loss patients in Japan
Source: Sci Rep. 2019 Aug 19;9:11976. doi: 10.1038/s41598-019-47141-4 (PMC6700179; doi:10.1038/s41598-019-47141-4)
Supplement: Supplementary file 1 — Table S1, Table S2, Figure S1, Figure S2, Figure S3, Figure S4, Figure S5 [file 41598_2019_47141_MOESM1_ESM.pdf]

Comprehensive analysis of syndromic hearing loss patients in Japan.

Supplementary information

Michie Ideura, Shin-ya Nishio, Hideaki Moteki, Yutaka Takumi, Maiko Miyagawa, Teruyuki Sato, Yumiko Kobayashi, Kenji Ohyama, Kiyoshi Oda, Takamichi Matsui, Tsukasa Ito, Hiroshi Suzumura, Kyoko Nagai, Shuji Izumi, Nobuhiro Nishiyama, Manabu Komori, Kozo Kumakawa, Hidehiko Takeda, Yoko Kishimoto, Satoshi Iwasaki, Sakiko Furutate, Kotaro Ishikawa, Masato Fujioka, Hiroshi Nakanishi, Jun Nakayama, Rie Horie, Yumi Ohta, Yasushi Naito, Mariko Kakudo, Hirofumi Sakaguchi, Yuko Kataoka, Kazuma Sugahara, Naohito Hato, Takashi Nakagawa, Nana Tsuchihashi, Yukihiko Kanda, Chiharu Kihara, Tetsuya Tono, Ikuyo Miyanohara, Akira Ganaha, Shin-ichi Usami

Supplementary Table S1. Clinical features and genetic analysis results for all BO/BOR syndrome patients.

Proband	Type	Hearing loss	Preauricular pits	Cervical fistula	Malformation			Renal anomaly	Variant
					Inner ear	Middle ear	External ear		
JHLB6679	typical	+	+	-	+	+	+	-	*EYA1* : c. [489T>G];[=]
JHLB346	typical	+	+	+	NA	NA	NA	NA	*EYA1* : c. [790C>T];[=]
#4107	typical	+	+	NA	+	+	NA	NA	*EYA1* : c. [823C>T];[=]
JHLB2279	typical	+	+	+	+	+	NA	-	*EYA1* : c. [823C>T];[=]
JHLB4689	typical	+	+	+	NA	+	NA	NA	*EYA1* : c. [867+5G>A];[=]
JHLB2922	typical	+	+	+	+	+	NA	NA	*EYA1* : c. [982C>T];[=]
JHLB3360	typical	-	+	+	-	NA	NA	+	*EYA1* : c. [982C>T];[=]
JHLB2645	typical	+	+	+	+	+	NA	NA	*EYA1* : c. [1155_1156delAT];[=]
#4361	typical	+	+	+	+	NA	NA	NA	*EYA1* : c. [1187A>G];[=]
JHLB2233	typical	+	+	-	+	+	-	NA	*EYA1* : c. [1220G>A];[=]
JHLB4043	typical	+	+	-	+	+	+	NA	*EYA1* : CNV
JHLB660	typical	+	+	NA	+	+	-	NA	*SIX1* : c. [519G>C];[=]
JHLB113	typical	+	+	+	NA	NA	NA	-	not identified
JHLB643	typical	+	+	-	+	+	NA	NA	not identified
JHLB4200	typical	+	+	-	NA	NA	+	+	not identified
JHLB4293	typical	+	+	-	NA	+	+	-	not identified
JHLB3868	atypical	+	+	-	+	NA	NA	NA	*EYA1* : c. [790C>T];[=]
#371	atypical	+	+	-	NA	NA	NA	NA	*EYA1* : c. [867+5G>A];[=]
JHLB2062	atypical	+	+	NA	+	NA	NA	-	*EYA1* : c. [982C>T];[=]
JHLB975	atypical	NA	+	NA	+	NA	NA	NA	*EYA1* : c. [1090C>T];[=]
JHLB3266	atypical	+	-	+	+	NA	NA	NA	*EYA1* : c. [1101-1G>A];[=]
#4079	atypical	+	+	-	+	NA	NA	NA	*EYA1* : c. [790C>T];[=]
JHLB2717	atypical	+	NA	+	+	NA	NA	NA	*EYA1* : c. [790C>T];[=]
JHLB237	atypical	+	+	NA	+	NA	NA	NA	not identified
JHLB266	atypical	+	+	NA	NA	NA	NA	NA	not identified
JHLB267	atypical	+	+	NA	NA	NA	NA	NA	not identified
JHLB344	atypical	+	+	NA	NA	NA	NA	NA	not identified
JHLB366	atypical	+	+	NA	NA	NA	NA	NA	not identified
JHLB394	atypical	+	+	NA	NA	NA	NA	NA	not identified
JHLB502	atypical	+	+	NA	NA	NA	NA	NA	not identified
JHLB576	atypical	+	+	NA	NA	NA	NA	NA	not identified
JHLB580	atypical	+	+	NA	NA	NA	NA	NA	not identified
JHLB591	atypical	+	+	NA	NA	NA	NA	NA	not identified
JHLB721	atypical	+	+	NA	NA	NA	NA	NA	not identified
JHLB769	atypical	+	+	NA	NA	NA	NA	NA	not identified
JHLB858	atypical	+	+	NA	NA	NA	NA	NA	not identified
JHLB883	atypical	+	+	NA	NA	NA	NA	NA	not identified
JHLB887	atypical	+	+	NA	NA	NA	NA	NA	not identified
JHLB1020	atypical	+	+	NA	NA	NA	NA	NA	not identified
JHLB2068	atypical	+	+	NA	NA	NA	NA	NA	not identified
JHLB2208	atypical	+	-	-	NA	NA	+	NA	not identified
JHLB2637	atypical	+	+	NA	NA	NA	NA	NA	not identified
JHLB2650	atypical	+	NA	+	NA	NA	NA	NA	not identified
JHLB2683	atypical	+	+	NA	NA	NA	NA	NA	not identified
JHLB2885	atypical	+	+	NA	NA	NA	NA	NA	not identified
JHLB2934	atypical	+	+	NA	+	NA	NA	NA	not identified
JHLB3038	atypical	+	+	NA	NA	NA	NA	NA	not identified
JHLB3298	atypical	+	+	NA	NA	NA	NA	NA	not identified
JHLB3301	atypical	+	+	NA	NA	NA	NA	NA	not identified
JHLB3781	atypical	+	+	NA	NA	NA	NA	NA	not identified
JHLB4120	atypical	+	+	NA	NA	NA	NA	NA	not identified
JHLB4160	atypical	+	+	NA	NA	NA	NA	NA	not identified
JHLB4312	atypical	+	+	NA	NA	NA	NA	NA	not identified
JHLB4366	atypical	+	+	NA	NA	NA	NA	NA	not identified
JHLB4491	atypical	+	+	NA	NA	NA	NA	NA	not identified
JHLB4603	atypical	+	+	NA	NA	NA	NA	NA	not identified
JHLB4709	atypical	+	+	NA	NA	NA	NA	NA	not identified
JHLB4730	atypical	+	+	NA	NA	NA	NA	NA	not identified
JHLB4952	atypical	+	+	NA	NA	NA	NA	NA	not identified

The reference cDNA sequencse NM172060 for *EYA1* , NM005982 for *SIX1* .

Supplementary Table S2. Novel variants identified in this study and the evidence of pathogenecity classification.

patient	gene	nucleotide change	Aminoacid change	SIFT	PP2	LRT	Mutation Taster	Mutation assessor	CADD	EXAC	GnomAD	Criteria of ACMG guideline	Evidence
JHLB-6679	*EYA1*	c.[489T>G];[=]	p.[Y163X];[=]	.	.	D(0)	A(1)	.	37	.	.	pathogenic	PVS1, PM2, PP1,PP3
JHLB-975	*EYA1*	c.[1090C>T];[=]	p.[Q364X];[=]	.	.	D(0)	A(1)	.	43	.	.	pathogenic	PVS1, PM2, PP3
JHLB-2645	*EYA1*	c.[1155_1156delAT];[=]	p.[L385fs];[=]	.	.	.	.	.	.	.	.	pathogenic	PVS1, PS2, PM2
JHLB-2091	*MITF*	c.[326dupC];[=]	p.[S109fs];[=]	.	.	.	.	.	.	.	.	pathogenic	PVS1, PM2, PP1
JHLB-1623	*MITF*	c.[389_399del];[=]	p.[Y130fs];[=]	.	.	.	.	.	.	.	.	pathogenic	PVS1, PM2, PP1
JHLB-1593	*MITF*	c.[550G>T];[=]	p.[E184X[;[=]	.	.	D(0)	A(1)	.	45	.	.	pathogenic	PVS1, PM2, PP1, PP3
JHLB-3463	*MITF*	c.[796G>T];[=]	p.[E266X];[=]	.	.	D(0)	A(1)	.	45	.	.	pathogenic	PVS1, PM2, PP1, PP3
JHLB-175	*SOX10*	c.[400_417del];[=]	p.[L134fs];[=]	.	.	.	.	.	.	.	.	pathogenic	PVS1, PS2, PM2
JHLB-1632	*SOX10*	c.[426G>C];[=]	p.[W142C];[=]	D(0)	D(1)	D(0)	D(1)	H(4.335)	29.4	.	.	likely pathogenic	PM2, PM5, PM6, PP3
JHLB-2550	*EDNRB*	c.[223delG];[=]	p.[D75fs];[=]	.	.	.	.	.	.	.	.	pathogenic	PVS1, PM2, PP1
JHLB-3591	*PAX3*	c.[318delC];[=]	p.[P106fs];[=]	.	.	.	.	.	.	.	.	likely pathogenic	PVS1, PM2
JHLB-4270	*SOX10*	c.[781_793del];[=]	p.[R261fs];[=]	.	.	.	.	.	.	.	.	likely pathogenic	PVS1, PM2
JHLB-3480	*SOX10*	c.[859delT];[=]	p.[S287fs];[=]	.	.	.	.	.	.	.	.	pathogenic	PVS1, PS2, PM2
JHLB-1192	*COL2A1*	c.[3198_3206del];[=]	p.[1066_1069del];[=]	.	.	.	.	.	.	.	.	likely pathogenic	PM2, PM4, PP1, PP4
JHLB-4194	*COL11A1*	c.[1737+2T>C];[=]		.	.	.	D(1)	.	24.6	.	.	pathogenic	PVS1, PM2, PP1, PP3
JHLB-4190	*COL11A1*	c.[3117__3152del];[=]	p.[1039_1051del];[=]	.	.	.	.	.	.	.	.	likely pathogenic	PS2, PM2, PP3
JHLB-2582	*OPA1*	c.[892A>C];[=]	p.[S298R];[=]	D(0)	D(1)	D(0)	D(1)	H(4.335)	27	.	.	likely pathogenic	PM2, PM5, PP3, PP4

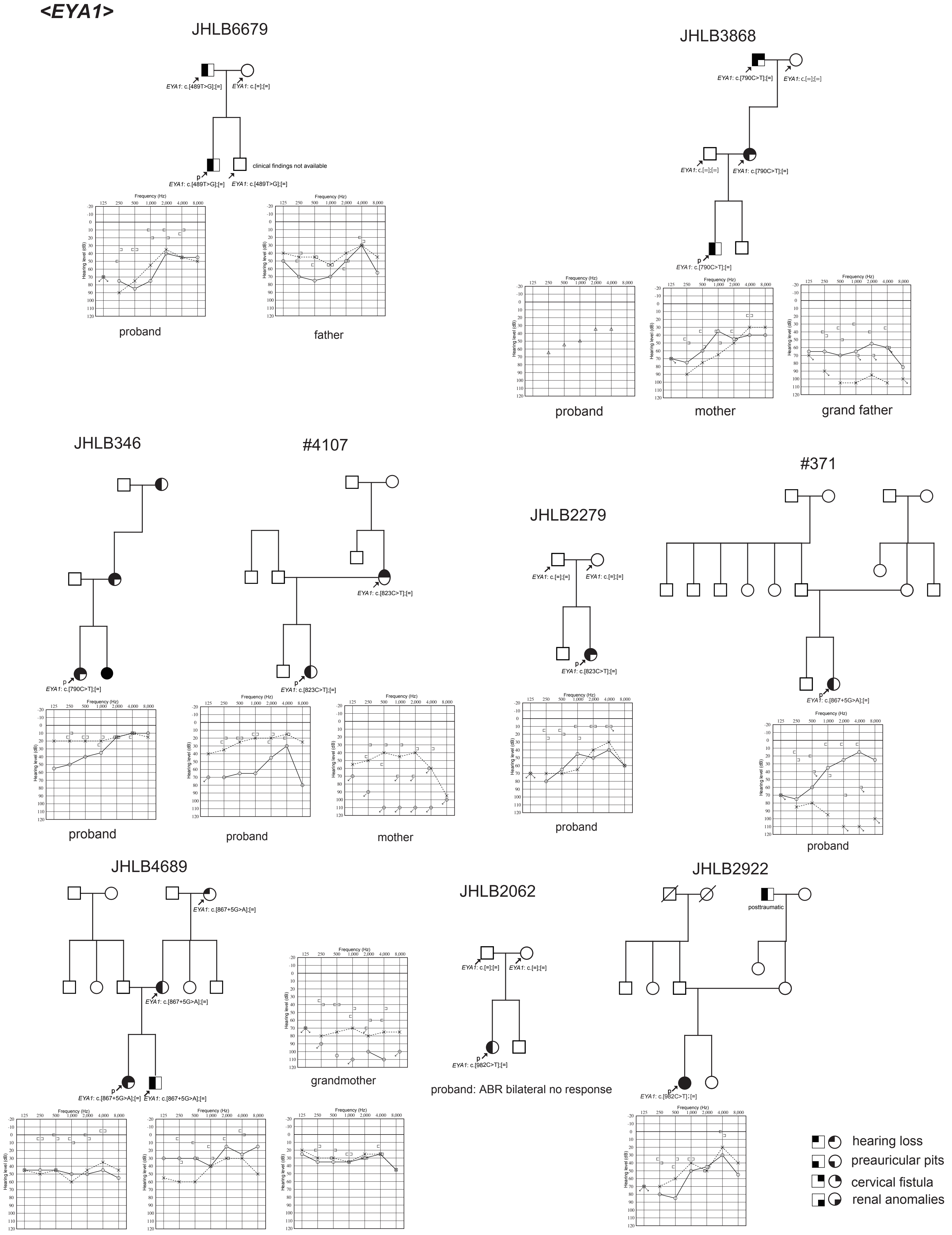

Supplementary figure1. The pedigrees and audiograms of BOR syndrome patients.

The reference cDNA sequence NM172060 for *EYA1*.

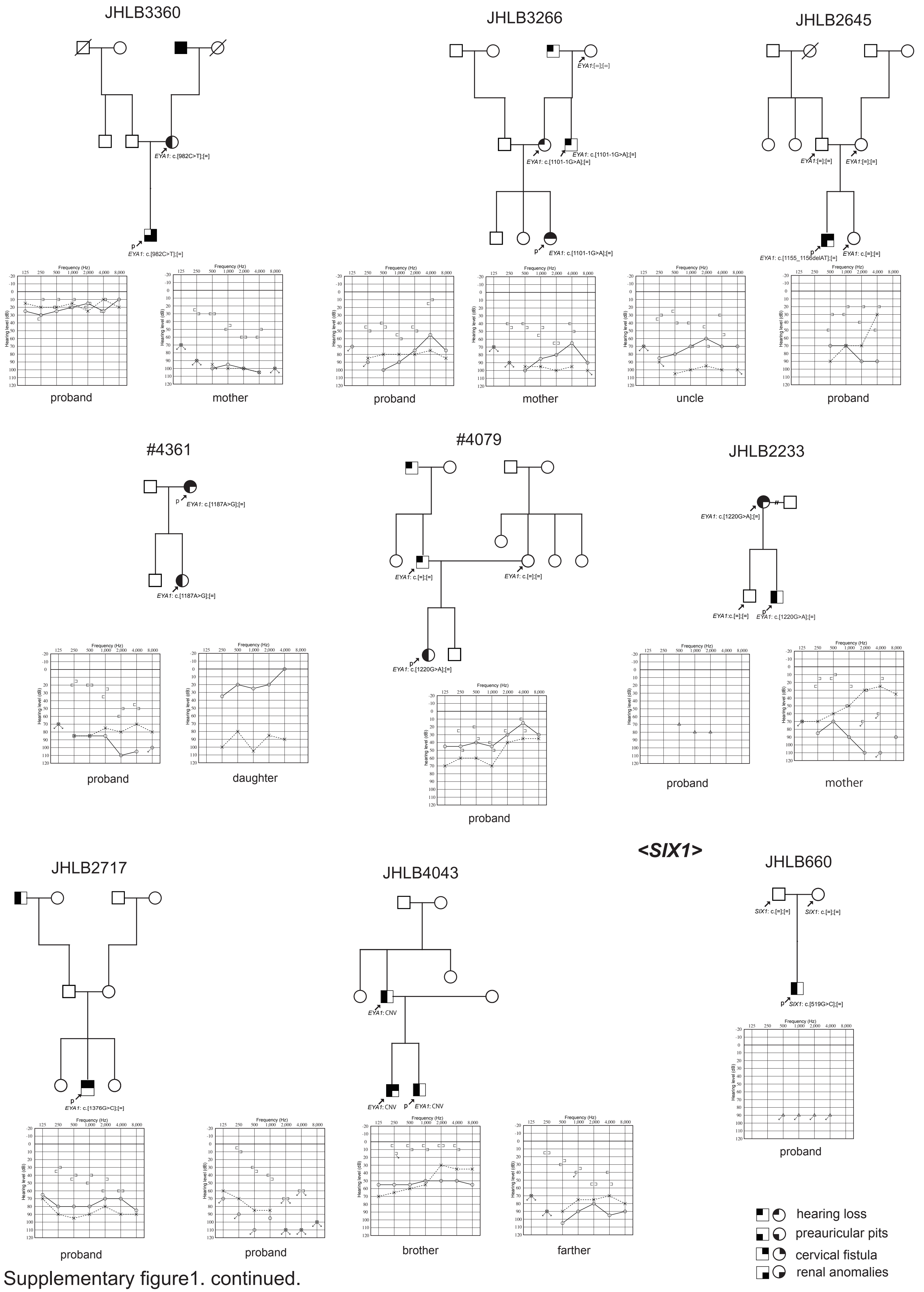

Supplementary figure1. continued.

The reference cDNA sequences NM172060 for *EYA1*, NM005982 for *SIX1*.

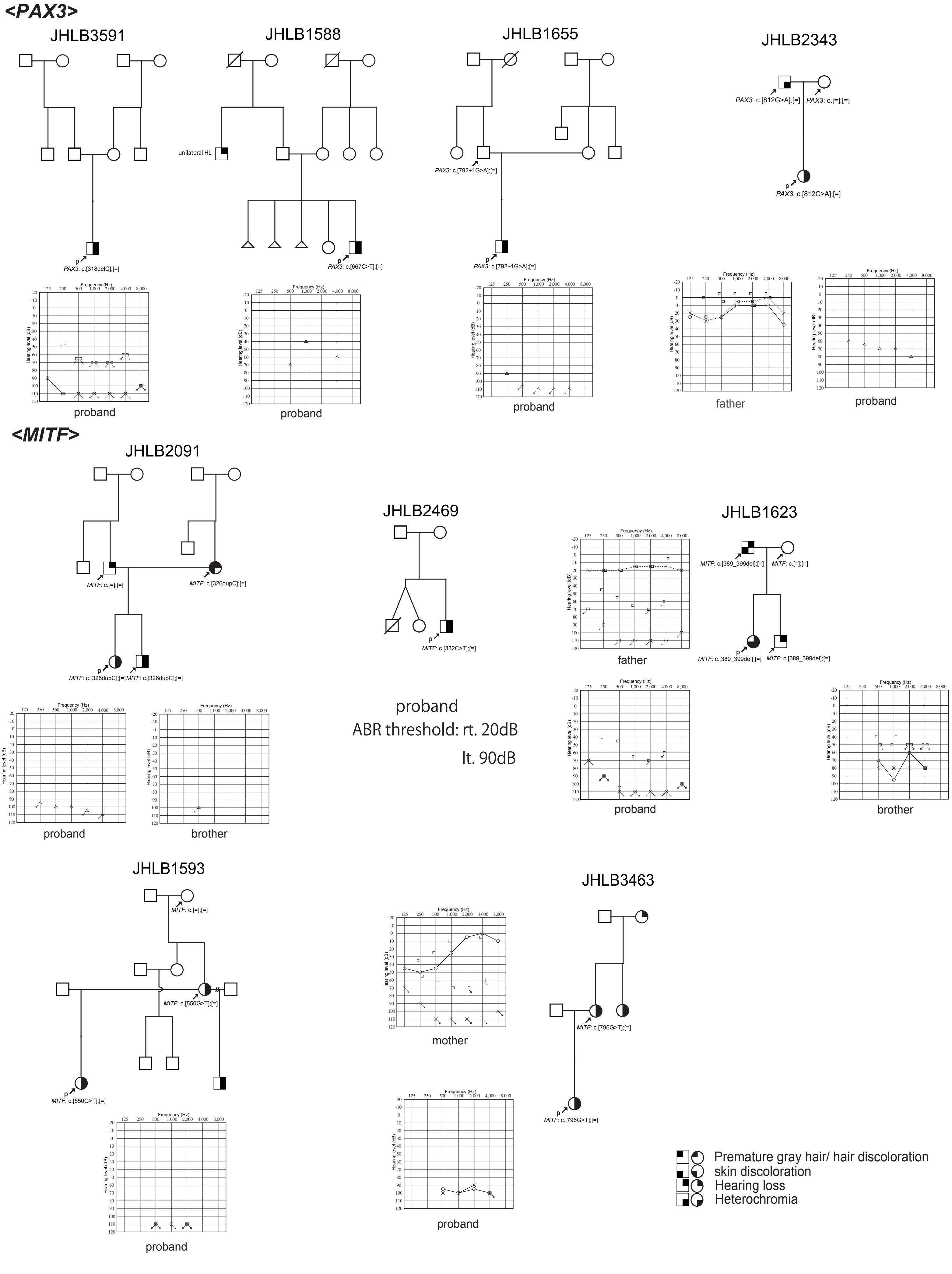

Supplementary Figure S2. The pedigrees and audiograms of Waardenburg syndrome patients.

The reference cDNA sequences NM181457 for *PAX3*, NM000248 for *MITF*.

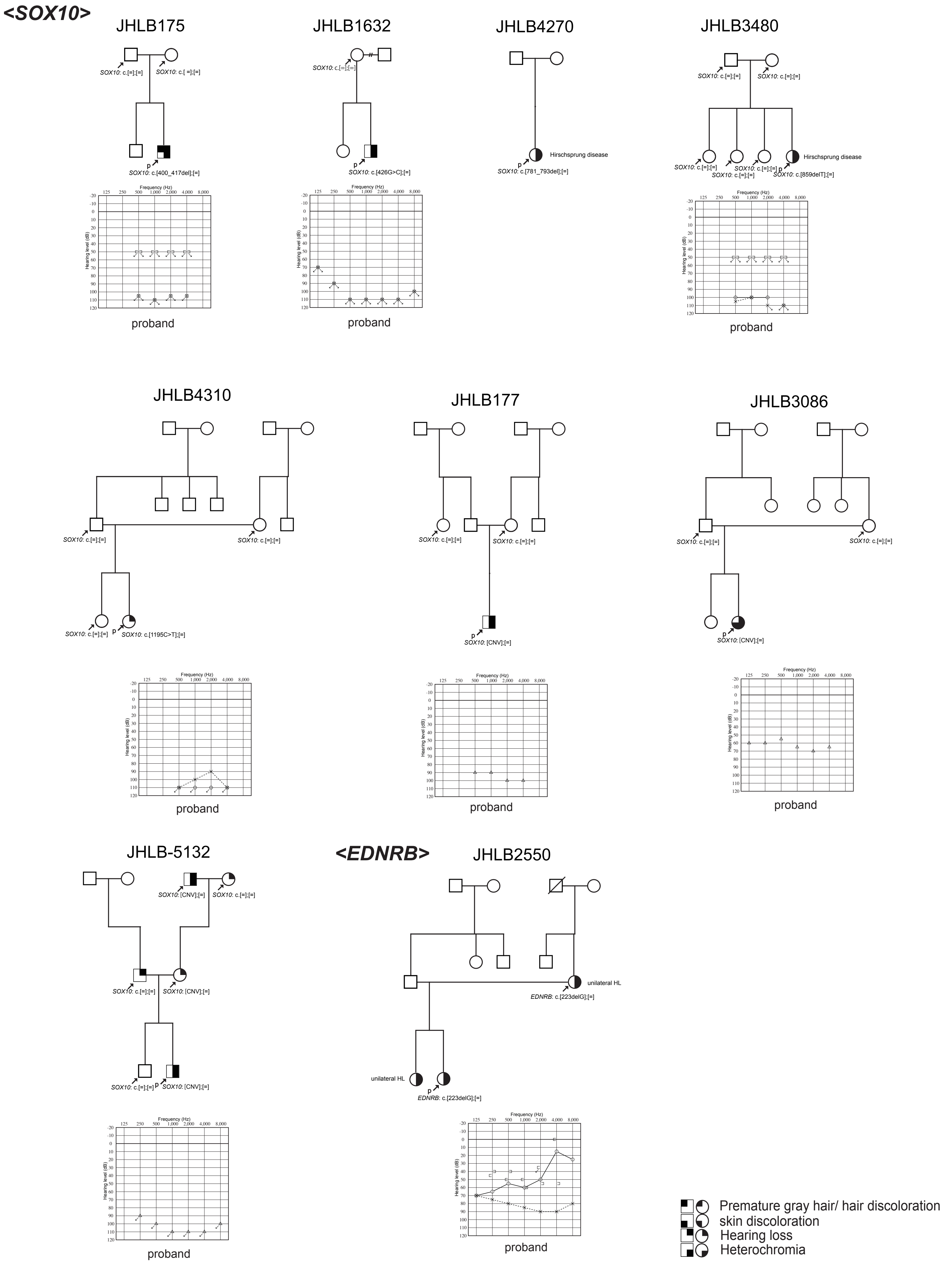

Supplementalry Figure S2. continued.

The reference cDNA sequences NM006941 for *SOX10*, NM000115 for *EDNRB*.

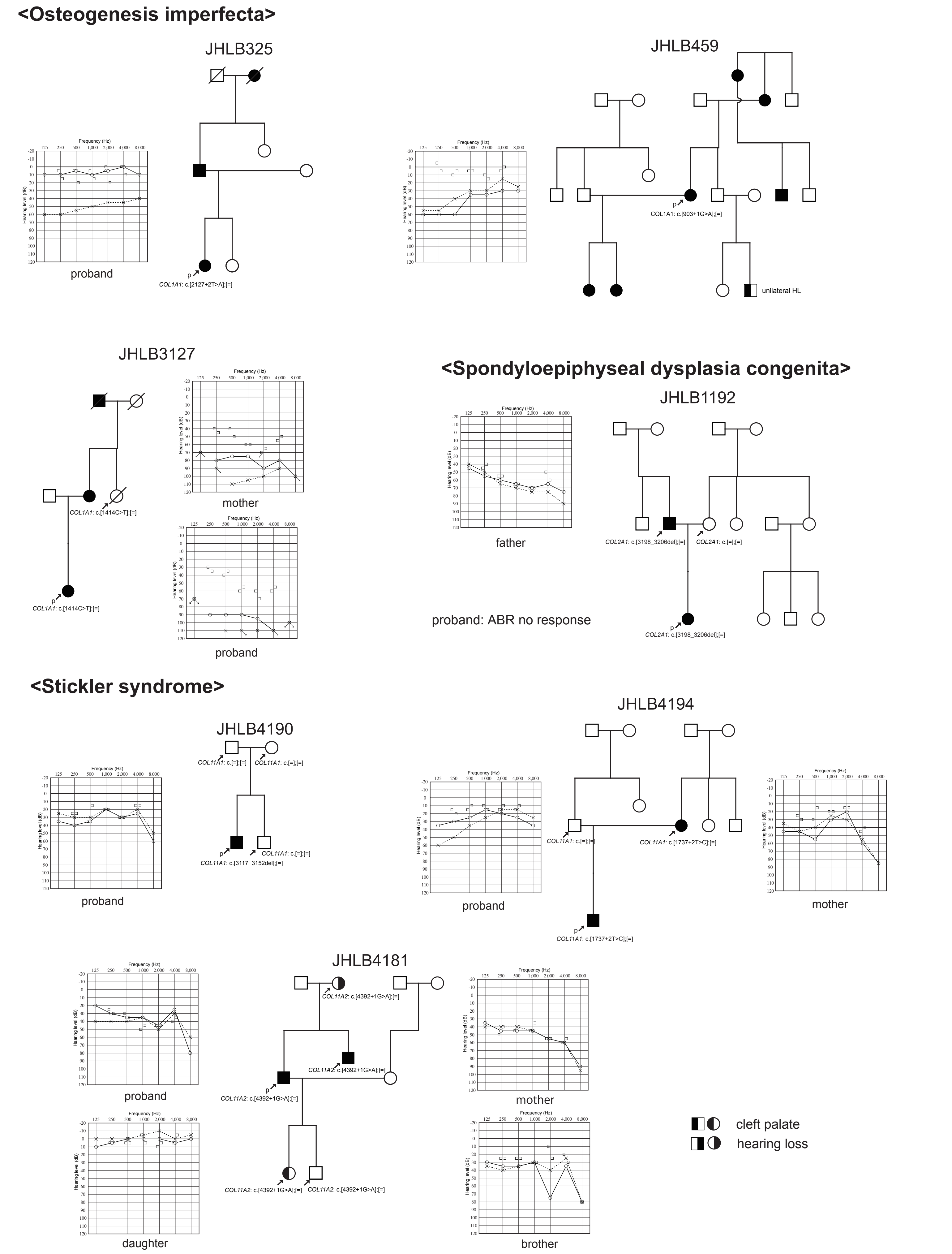

Supplemantary figure3. The pedigrees and audiograms of other syndromic hearing loss patients.

The reference cDNA sequences NM000088 for *COL1A1*, NM001844 for *COL2A1*, NM001854 for *COL11A1*, NM080680 for *COL11A2*.

<CHARGE syndrome>

JHLB448

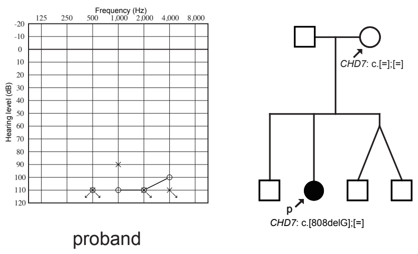

<Jervell Lange-Nielsen syndrome>

JHLB4860

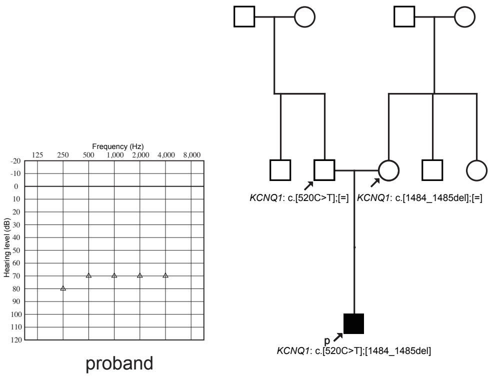

<Auditory neuropathy with optic atrophy>

JHLB2582

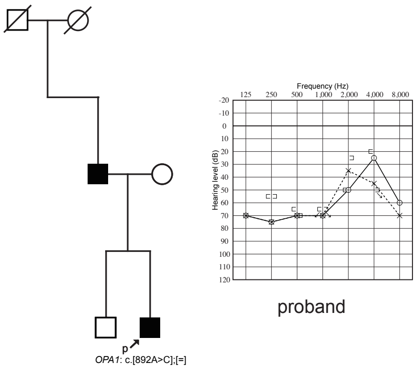

Supplemantal figure3. continued.

The reference cDNA sequences NM017780 for *CHD7*, NM000218 for *KCNQ1*, NM015560 for *OPA1*.

<Pendred syndrome>

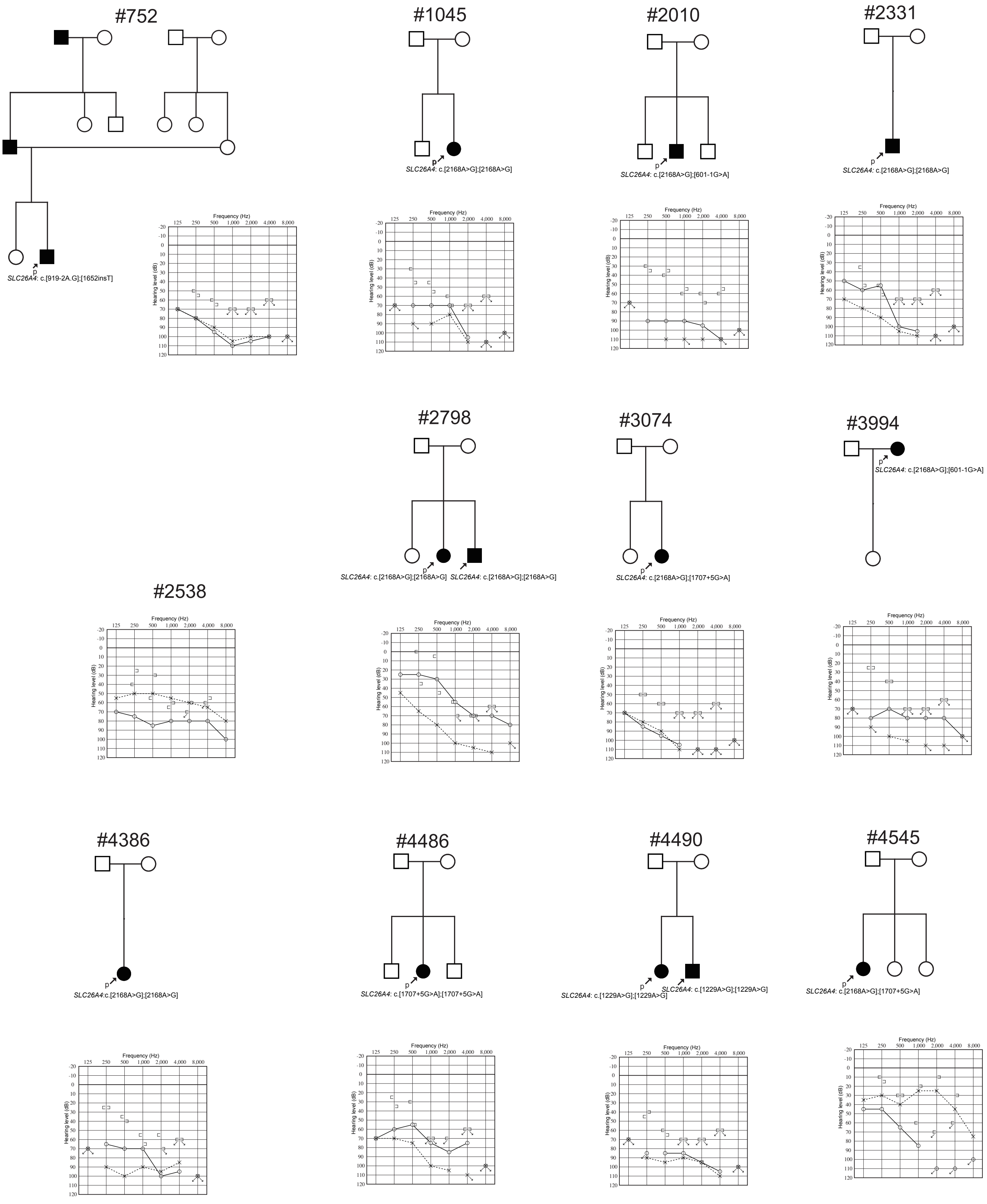

Supplementary figure4. The pedigrees and audiograms of Pendred syndrome patients.

The reference cDNA sequence NM000441 for *SLC26A4*.

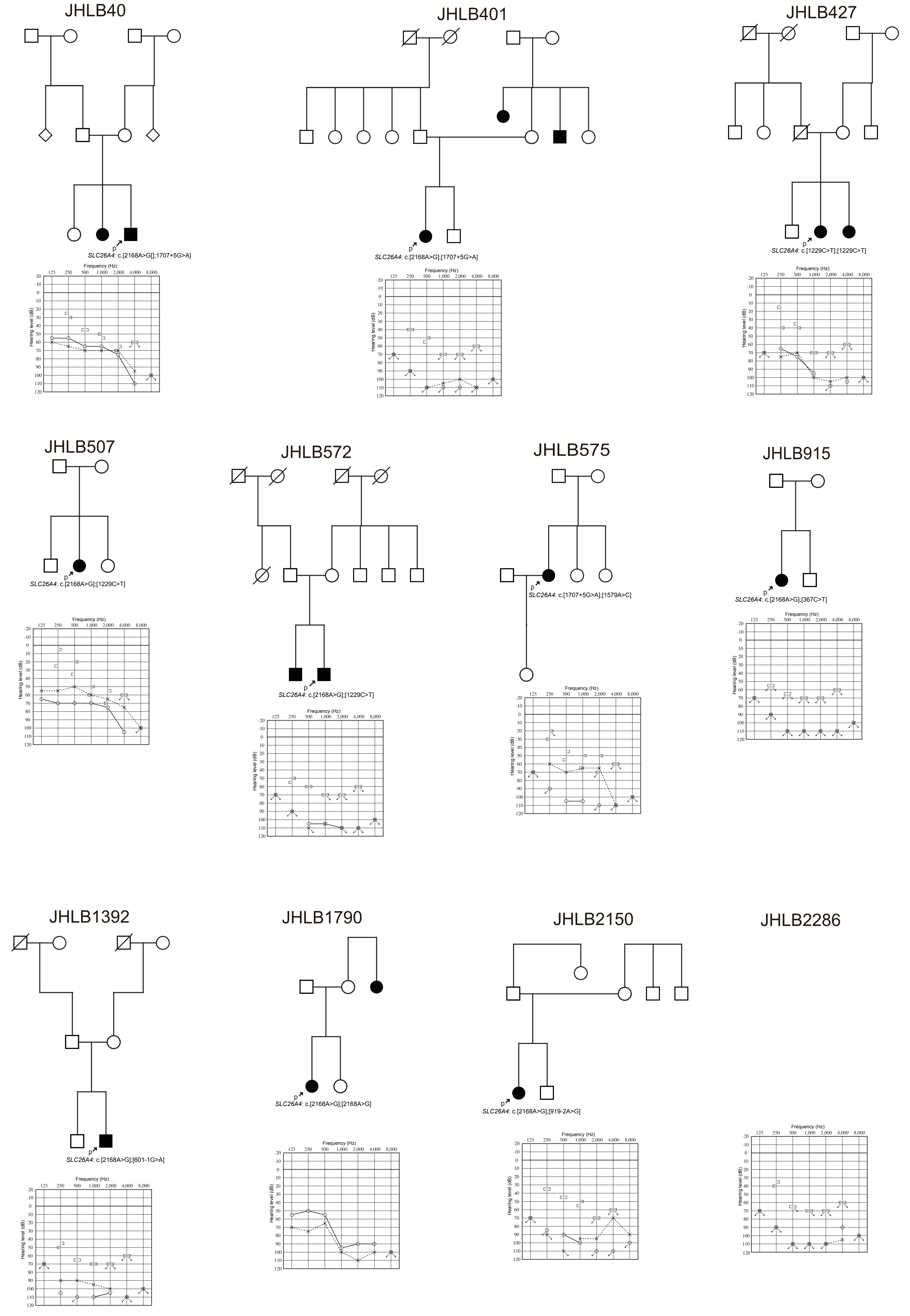

Supplementary Figure S4.continued.

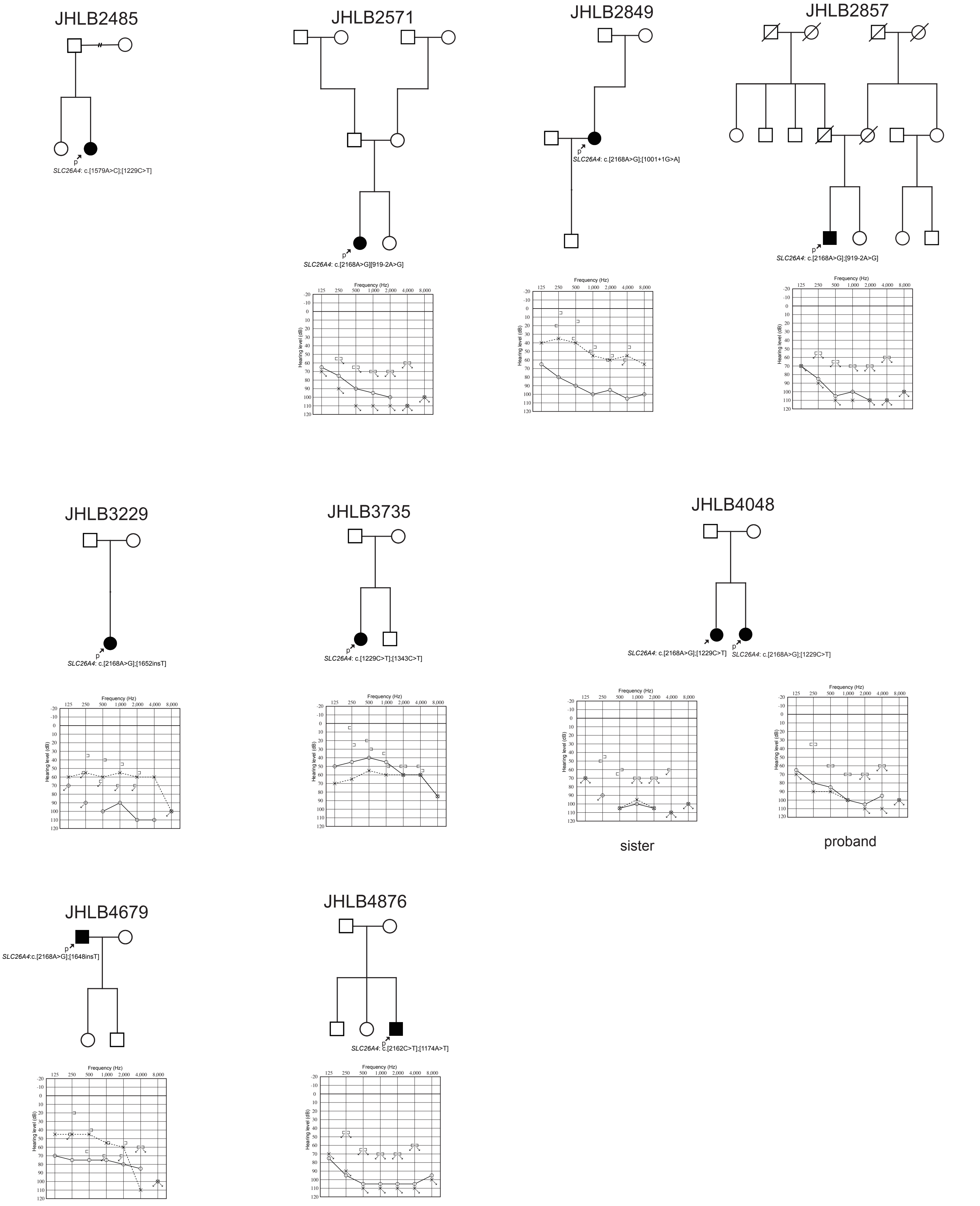

Supplementary figure4. Comtinued.

Supplementary Figure S5. W-index of Japanese control population in the previous report.

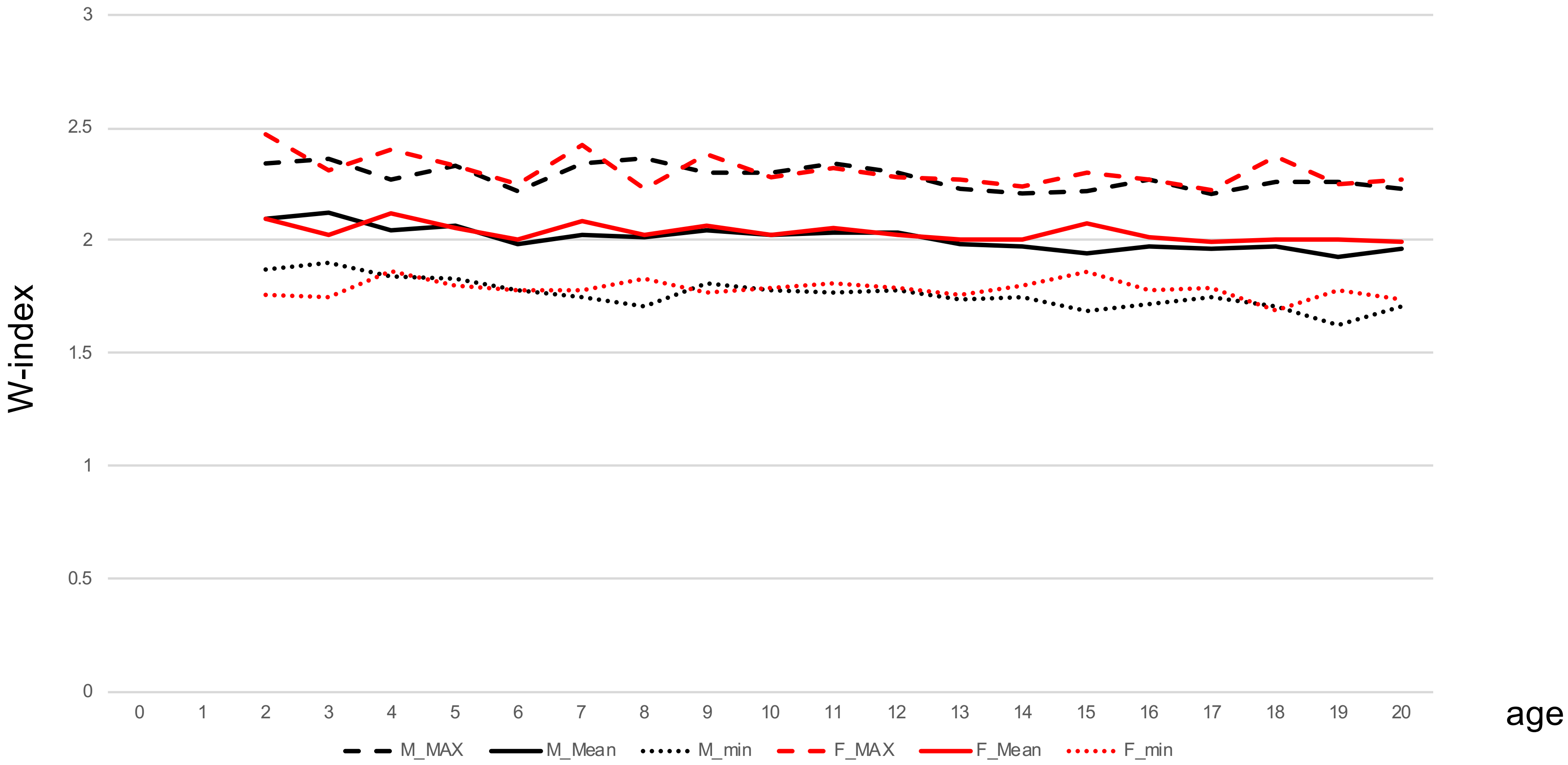

M: Male, F: Female

Graph was plotted from the eye distance data in reference 47 (in Japanese).
